# Phenolic Compounds and Expression of *4CL* Genes in Silver Birch Clones and Pt4CL1a Lines

**DOI:** 10.1371/journal.pone.0114434

**Published:** 2014-12-11

**Authors:** Suvi Sutela, Terhi Hahl, Heidi Tiimonen, Tuija Aronen, Tiina Ylioja, Tapio Laakso, Pekka Saranpää, Vincent Chiang, Riitta Julkunen-Tiitto, Hely Häggman

**Affiliations:** 1 Department of Biology, University of Oulu, Oulu, Finland; 2 The Finnish Border Guard, Border and Coast Guard Academy, Imatra, Finland; 3 Finnish Forest Research Institute, Eastern Finland Regional Unit (Punkaharju Unit), Punkaharju, Finland; 4 Finnish Forest Research Institute, Southern Finland Regional Unit (Vantaa Unit), Vantaa, Finland; 5 Forest Biotechnology Group, Department of Forestry and Environmental Resources, North Carolina State University, Raleigh, North Carolina, United States of America; 6 Department of Biology, University of Eastern Finland, Joensuu, Finland; UCLM, Spain

## Abstract

A small multigene family encodes 4-coumarate:CoA ligases (4CLs) catalyzing the CoA ligation of hydroxycinnamic acids, a branch point step directing metabolites to a flavonoid or monolignol pathway. In the present study, we examined the effect of antisense *Populus tremuloides 4CL* (*Pt4CL1*) to the lignin and soluble phenolic compound composition of silver birch (*Betula pendula*) Pt4CL1a lines in comparison with non-transgenic silver birch clones. The endogenous expression of silver birch *4CL* genes was recorded in the stems and leaves and also in leaves that were mechanically injured. In one of the transgenic Pt4CL1a lines, the ratio of syringyl (S) and guaiacyl (G) lignin units was increased. Moreover, the transcript levels of putative silver birch *4CL* gene (*Bp4CL1*) were reduced and contents of cinnamic acid derivatives altered. In the other two Pt4CL1a lines changes were detected in the level of individual phenolic compounds. However, considerable variation was found in the transcript levels of silver birch *4CL*s as well as in the concentration of phenolic compounds among the transgenic lines and non-transgenic clones. Wounding induced the expression of *Bp4CL1* and *Bp4CL*2 in leaves in all clones and transgenic lines, whereas the transcript levels of *Bp4CL3* and *Bp4CL4* remained unchanged. Moreover, minor changes were detected in the concentrations of phenolic compounds caused by wounding. As an overall trend the wounding decreased the flavonoid content in silver birches and increased the content of soluble condensed tannins. The results indicate that by reducing the *Bp4CL1* transcript levels lignin composition could be modified. However, the alterations found among the Pt4CL1a lines and the non-transgenic clones were within the natural variation of silver birches, as shown in the present study by the clonal differences in the transcripts levels of *4CL* genes, soluble phenolic compounds and condensed tannins.

## Introduction

Phenolic compounds form a diverse group of secondary metabolites exhibiting important roles in plant development and environmental adaptation [1]–[3]. A benzene ring attached to at least one hydroxyl group is a common characteristic of phenolic compounds, mostly derived from cinnamic acid, generated at the first step of the general phenylpropanoid pathway. Carbon flow through the general phenylpropanoid route is extensive as the hydroxycinnamic acids are utilized in the monolignol biosynthetic pathway, giving rise to the second most abundant organic compound on earth, lignin. The accumulation of lignin in secondary cell walls of vascular plants provides mechanical strength, protection against pathogens and herbivores, and enables the essential transportation of water and solutes. Flavonoids, hydroxycinnamic acids, phenolic glycosides, and condensed tannins (CTs) represent other intensively studied phenolics having functions in allelopathy, pollen fertility, auxin transport, pollinator attraction and in defence against pathogens, pests, UV-B radiation, and oxidative damage [1]–[3].

Reflecting the divergent roles of phenolic compounds, the general phenylpropanoid route can be activated by abiotic and biotic stress factors, such as cold, ozone, CO_2_, light, pathogens, and herbivores [4]. In addition, the phenylpropanoid route is triggered by mechanical wounding as upon damage the wound site needs to be sealed in prevention of dehydration and possible pathogen infections. At the wound site the cell walls are first strengthened by crosslinking proteins. Phenylpropanoid derivatives are required in the subsequent step, lignification and suberization of the cell walls [5]. Furthermore, mechanical wounding can induce production of phenolic compounds which are generally considered to interact in the defence responses. However, similar to induced defences caused by herbivory response, also the accumulation of phenolic secondary metabolites is species dependent [6], [7].

The general phenylpropanoid route is initiated by the deamination of phenylalanine by phenylalanine ammonia-lyase (PAL; EC 4.3.1.5). In the following step cinnamic acid can be converted to *p*-coumaric acid by cinnamic acid 4-hydroxylase (C4H; EC 1.14.13.11). Finally, as the last step of the general phenylpropanoid route, 4-coumarate:CoA ligase (4CL; 6.2.1.12) catalyzes the formation of *p*-coumaroyl-CoA. In addition to *p*-coumaric acid, the 4CL isoforms are able to utilize a variety of hydroxycinnamic acid derivatives as substrates, and positive connection has been found between the substrate diversity of 4CLs and the extent of gene family expansion [8]. However, the *4CL* gene families are relative small, for instance in Arabidopsis the number of *4CLs* is three [9]–[11]. The angiosperm *4CLs* are categorized to two classes: class I and class II [9], [12], [13]. Most of characterized *4CLs* belong to class I, the members of which are generally considered to function in the lignin biosynthesis [9], [13]–[20]. In addition, the *4CL* gene families often include 4CLs having distinct substrates such as sinapate [12], [21] and *4CL*-like genes, generally considered neither to be connected with lignification nor flavonoid biosynthesis [9], [10], [22], [23].

In the phenylpropanoid route, 4CL is considered as a branching point at which the metabolic fluxes are directed either to flavonoid or monolignol biosynthetic pathway. The monolignol biosynthetic pathway produces lignin monomers, coniferyl -, sinapyl - and *p*-coumaryl alcohol, in cytoplasm and subsequent polymerisation of monolignols with polysaccharides occurs in the plant cell wall. In hardwoods lignin is composed of guaiacyl (G), syringyl (S) and *p*-hydroxyphenyl (H) units having different methylation degrees which results in varying linkage types in the polymer. Hence, the structural complexity and chemical solubility of lignin depend on its monolignol composition. The composition and content of lignin can be altered by modifying the expression of monolignol biosynthetic pathway genes [11], [24]. By reducing the expression of particular *4CL* genes, the lignin content has been decreased in Arabidopsis [11], [16], *Populus tremuloides* [25], [26], *P*. *tomentosa* [27], [28], *P*. *tremula* × *alba* [29], *P*. *trichocarpa* [30], *Nicotiana tabacum* [31], rice (*Oryza sativa*) [20], and *Pinus radiata* [32]. In addition to the lignin content, also the lignin composition [16], [28], [29], [32], [33] and concentration of phenolics [11], [20], [25], [28], [29], [32], [34] have been shown to alter as a result of reduced *4CL* expression.

Silver birch (*Betula pendula* Roth) has been under active research in Finland due to its economic importance and central role in the boreal forest ecosystems [35]. Besides the conventional breeding methods, the modern molecular methods have been shown to be applicable for silver birch [35], [36]. Furthermore, the possible environmental risks associated to genetically modified (GM) trees, have been studied with silver birch lines having introduced traits targeted to modify the lignin characteristics [37]–[40] and disease resistance [41]–[46]. In general, modest pleiotropic effects have been detected in the studies with GM silver birch lines. In the present work phenolic compounds and the expression of endogenous *4CL*s are studied in four silver birch clones. Silver birch lines expressing antisense quaking aspen (*Populus tremuloides* L.) *Pt4CL1* gene, were generated and characterized and the effect of mechanical wounding on soluble phenolic compounds and *4CL* gene expression was determined.

## Materials and Methods

### Generation of Pt4CL1a silver birch lines

Three transgenic lines representing the silver birch (*Betula pendula* Roth) genotypes A, E5382, and E5396 were generated by transformation with *Pt4CL1* (AF041049), the gene derived from quaking aspen (*P*. *tremuloides*). The clone A represents the progeny from the crosses between silver birch lines of southern Finnish origin, E1970 (Kangasala) and E1980 (Nummi-Pusula). E5382 and E5396 were cultivated in the clonal archive in Punkaharju (61°49′ N; 29°18′ E).

The *Pt4CL1* expressing silver birch lines were generated by the means of biolistic transformation with the PDS-1000/He devise (Bio-Rad Laboratories Inc., Hercules CA) using *in vitro* stem pieces as explants as described in Valjakka *et al.* [36]. The gene construct pRT9/35S-*PtCOMT* [47] was used as a backbone for the construction of pRT99/35S-*Pt4CL1*-a plasmid vector. The pRT9/35S-*PtCOMT* contained the neomycin phosphotransferase II (*nptII*) gene under the control of the CaMV 35S promoter and the *PtCOMT* fragment followed by the NOS terminator driven also by the CaMV 35S promoter. The pRT99/35S-*Pt4CL1*-a (S1 Figure in S1 File) was generated by substituting the *PtCOMT* sequence with the *Pt4CL1* using the *Xba*I and *Bam*HI restriction sites. The 1.9 kb long fragment of *Pt4CL1* was ligated to the promoter and NOS terminator in antisense orientation and the accuracy of construct was confirmed by sequencing. The selection of transformed silver birch material was based on the usage of 100–200 mg/L kanamycin (kan) in the Woody Plant Medium (WPM, [48]) after a week's cultivation on antibiotic free WPM [47]. The regeneration of individual silver birch lines, as well as rooting, was accomplished on the WPM as described in Aronen *et al.* [47].

The integration of the gene construct and the expression of transgenes were confirmed using Southern and Northern blot analysis. The DNA extraction was performed as described in Valjakka *et al.* [36], and Southern blot analysis as described in Aronen *et al.* [47]. The restriction enzymes *Xba*I and *Bam*HI were used in the digestion of 15 µg genomic DNA. The digoxigenin-11-dUTP labelled probes specific for *nptII* and *Pt4CL1* were generated with PCR reaction described in Aronen and Häggman [49] with primers presented in S1 Table in S2 File. The procedure for total RNA isolation and for the Northern blot can be found in Aronen *et al.* [47].

### Characterization of the Pt4CL1a silver birch lines

The first experimental set-up included the transgenic lines A1, A2, A5 and E5382/3 derived from clones A and E5382, respectively. The second experimental set-up consisted of clone E5396 and the transgenic line E5396/4. Both experiments were conducted at the greenhouse of Finnish Forest Research Institute Punkaharju Unit under standard greenhouse conditions and natural light conditions.

In the first experimental set-up, the potted plants of A, A1, A2, A5, E5382 and E5382/3 were placed into four replicates in June where 80 plants were placed in randomly assigned design. To avoid potential edge effects they were surrounded by additional plants not used as experimental material. The number of individual plants of clones E5382 and A and the transgenic lines A1, A2, and A5 was equal, that is, 13 individuals in the three replicates and 12 individuals in the fourth. The number of plants of transgenic line E5382/3 was 11 in the three replicates and 10 in the fourth. Furthermore, the three replicates included three, and the fourth replicate 13 additional birches surrounding the studied plants.

The morphology, growth characteristics and phenology of the silver birches were recorded for two growing seasons after which nine individual silver birches per line/clone were selected to be monitored and grown for additional third growing season. The stem and leaf samples for lignin and Northern blot analyses were collected during second growing season. The leaves were collected below the latest shoot and mature leaves in short shoots at the base of lateral shoots were used. Part of the collected leaves was used in experiments with herbivours larvae.

The experiment with clone E5396 and transgenic line E5396/4 consisted of two replicates. Each experiment was composed of 30 potted individual plants representing clone E5396 and line E5396/4 and they were organized into parallel, randomly assigned design. The two replicate experiments contained altogether 120 plants and surrounded by additional birches not used as experimental material. The experiment started in August. At the end of the second growing season, final growth parameters were recorded, and samples taken for analyses.

The Klason lignin measurements were conducted for 2-year-old stem samples representing all birch clones and lines as described in Aronen *et al.* [47]. Besides, the determination of S and G moieties was conducted with 2-year-old stem samples, however, without samples from the clone E5396 and the transgenic line E5396/4. The procedure and the chromatographic conditions were carried out as described by Tiimonen *et al.* [37].

### Test with lepidopteran larvae

The food quality of leaves were examined by offering the leaves to larvae of three geometrids *Aethalura punctulata* Denis & Schiff., *Cleora cinctaria* Denis & Schiff., *Epirrita autumnata* Bork., and noctuid *Orthosia gothica* L. in a no-choice test. The adults of *A*. *punctulata*, *C*. *cinctaria*, and *O*. *gothica* were captured in south-eastern Finland and larvae of *E*. *autumnata* were collected in northern Finland. Larvae were reared on diet consisting of *B*. *pendula*. Of the four plants per replicate of clones A and E5382 and lines A1, A2, A5, and E5382/3 were randomly selected and leaves collected to the RGR experiment. The full-grown short shoot leaves were collected from the lateral shoots. Each larva was weighted and positioned on a leaf which was on a moist filter paper on a Petri dish of 9 cm of diameter. The experiment continued for 24 h which after the larvae were weighted again. For *A*. *punctulata*, *C*. *cinctaria* and *E*. *autumnata* the experiment was repeated using the same plant individuals (series I and II). The RGR values ([ln(end weight) - ln(initial weight)]/d) of larvae were excluded when no weight gain was recorded or when larvae had died/moulted or appeared otherwise unwell. In addition, when larvae had consumed whole or almost whole leaf or if the leaf had begun yellowing, values were not included in the analysis.

### Wounding experiment

The plant material consisted of four non-transgenic silver birch clones (A, R, E5382, E5396) and four transgenic lines (A1, A5, E5382/3, E5396/4). The non-transgenic clone R represents a progeny from a cross between registered silver birches V5411 and V5402. The silver birch material was multiplied at 21–22°C with light/dark photoperiod of 16 h/8 h (110–130 µmol m^−2^ s^−1^) on WPM supplemented with 2.2 µM 6-benzyladenine (BA) and 2.8 µM indole-3-acetic acid (IAA), and accompanied with 200 mg/L kan for the transgenic silver birch lines. The rooting of plantlets was conducted on WPM without plant growth regulators or antibiotics. The 5-week acclimatization period of plants took place at the Botanical Gardens of the University of Oulu on a mixture of Kekkilä Seedling Soil (Kekkilä Oy, Finland) and sand. The plants were transplanted in March and placed in a randomly assigned experimental design in the beginning of April. The number of individual plants in the experimental design varied per clone/line between 70 and 120.

The wounding of plants was conducted during April and May by mechanically crushing approximately 20% of the leaf margins of two adjacent leaves in each plant using pliers. The plants of similar heights were selected for the control and wounding treatments. The leaf plastochron index (LPI) of the two wounded leaves ranged from LPI 2 to LPI 4 when the first fully expanded leaf was considered as LPI 0. When counted from the plant apex the position of LPI 2–4 leaves ranged between the seventh and eleventh leaf, depending on the plant individual. The samples were taken immediately, and 1, 3, 12, 24, 72, and 168 hours after the wounding for the determination of transcript levels. For the analysis of phenolic compounds, the samples were collected 21 d after the wounding. At every sampling intact control samples were also collected. The stem heights and fresh weights of all plants and samples were recorded at sample collection. The average stem height (± SD) of clones/lines were 15.5±3.1, 16.8±3.6, 16.2±3.5, 17.9±4, 22.8±4, 14.5±5.1, 15.1±3, and 17.8±3.5 cm for A, A1, A5, E5382, E5382/3, E5396, E5396/4, and R, respectively.

### Isolation of full-length 4CL and 4CL-like genes

The sequencing of silver birch *4CL* genes was based on the EST data obtained from Helariutta and Kauppinen (University of Helsinki, Finland). The ESTs were aligned and searched against the National Center for Biotechnology Information (NCBI) database. The primers were designed for expected coding sequences (cds) of the four *4CL* genes with Primer3 [50], [51] and used in standard PCR runs or in additional runs with the SMART RACE cDNA Amplification Kit (Clontech Laboratories, Inc., Mountain View, California, US) in accordance with the manufacturer's instructions. The PCR products representing the full cds of putative *4CL* genes were produced with primers presented in S1 Table in S2 File and purified with Nucleo Spin Extract II Kit (Macherey-Nagel GmbH & Co. KG, Düren, Germany). Subcloning was conducted with TOPO TA Cloning (Invitrogen) and sequencing with the BigDye Terminator v3.1 Cycle Sequencing Kit (Applied Biosystems, Foster City, CA, USA) and the ABI PRISM 377 DNA sequencer (Perkin-Elmer, Wellesley, MA, USA).

### Phylogenetic analysis

The phylogenetic analyses were conducted using MEGA6 [52]. The cds of Manihot esculenta (Mes4CL1-4), Glycine max (Gm4CL1-9), Rubus idaeus (Ri4CL1-3), P. tremuloides (Pt4CL1 and Pt4CL2), Betula platyphylla (Bpl4CL1), and Betula luminifera (Bl4CL1) were used in phylogenetic analyses. In addition, a selection of the 4CL and 4CL-like acyl-CoA synthetase (ACS) genes of Arabidopsis, Populus trichocarpa and rice [23] were included into the analysis. All used sequences are presented in S5 Table in S3 File. The 4CL cds were aligned with MUSCLE [53]. The Model Selection feature was used to evaluate the substitution models for maximum-likelihood (ML) method. A phylogenetic tree was reconstructed using the ML algorithm [54] and Tamura 3-parameter substitution model [55] with the gamma distributed with invariant sites model. All codons were included, and partial deletion was used to positions containing missing data or gaps as recommended by Hall [56]. The confidence of ML trees was evaluated with bootstrap method using 500 replicates [57] and bootstrap values ranging between 70 and 100% were considered reliable [58].

### Determination of 4CL transcript levels

From leaf material the RNA was extracted using the protocol of Jaakola *et al.* [59], whereas from stems the RNA isolation was conducted with the GeneJET Plant RNA Purification Mini Kit (Thermo Scientific, Waltham, Massachusetts, USA) using the protocol for RNA purification from lignified, polyphenol-rich plant tissues. Before treating the RNA with DNase I (Thermo Scientific) RNA was characterized with agarose gel electrophoresis and ND-1000 UV-Vis Spectrophotometer (NanoDrop Technologies, Wilmington, USA). The RevertAid Premium Reverse Transcriptase (Thermo Scientific) was used in the synthesis of cDNA, which was diluted 1∶50 for the real-time RT-PCR runs. The real-time RT-PCR reactions of 14 µL consisted of 1 x LightCycler 480 SYBR Green I Master (Roche Applied Science, Penzberg, Germany), 0.5 µM of each primer (S1 and S2 Tables in S2 File) and 3.5 µL of cDNA sample and were run as duplicates with LightCycler 480 (Roche Applied Science). The PCR program consisted of incubation at 95°C for 5 min followed by 45 cycles: 10 s at 95°C, 10 s at 60°C and 10 s at 72°C. The melting curve analysis of the LightCycler 480 software and sequencing of the real-time RT-PCR products, using the ABI PRISM 377 DNA sequencer (Perkin-Elmer) and chemistry of the BigDye Terminator v3.1 (Applied Biosystems) were used to confirm the specificity of the primers. The alpha-tubulin (*Atub*, AJ279695) and putative protein phosphatase 2A regulatory subunit (*PP2A*, FJ667540) were used as reference genes with primers described in Sutela *et al.* [40]. Serial dilutions of pooled cDNA were used to create primer specific efficiencies for stem and leaf samples (S1 and S2 Tables in S2 File). The Abs Quant/2nd Derivative Max for All Samples Analysis of Lightcycler 480 Software release 1.5.0 SP3 was utilized to generate the crossing point (Cq) and concentration values (S3 and S4 Tables in S3 File). If Cq could not be determined the concentration value was consider as 0. The performance of technical replicates was evaluated and, when necessary, samples were rerun (see S2 Figure and S3 Figure in S1 File for the coefficient variation, CV%, of technical replicates). The relative expression values were obtained using relative quantification with external standards (Roche Applied Science Technical Note No. LC 13/2001) and the relative expression represents the ratios of target and reference genes. For the determination of wounding response, the relative expression values were divided by the mean values of intact control leaves within each clone/line. The *Atub* and *PP2A* were utilized as reference genes as no trend was observed in the Cq values due to the wounding treatment (S4 Figure in S1 File).

### Analysis of soluble phenolic compounds and CTs

The leaf and stem samples were dried at 60°C for 48 h, after which they were stored at −20°C. The pooled samples of leaf (8 mg) were cut with a cork borer without the main and lateral veins and, in the case of wounded leaves, also without the damaged leaf area. Of each plant individual a pooled stem sample of 15 mg presenting only un-wounded plants was used in the analysis of phenolic compounds. The leaves and stems were homogenized with Precellys 24 homogenizer (Bertin Technologies, Montigny-le-Bretonneux, France) with 600 µL of methanol placed into each Precellys homogenization vial. The homogenization was conducted at 2800 g for 20 s followed by 15 min incubation on ice. The homogenization step was repeated (2800 g for 20 s) after which the samples were centrifuged at 19 000 g for 3 min. Supernatants were collected and the extraction was repeated three more times using 5 min incubations on ice. The combined supernatants were dried in a vacuum concentrator (Concentrator 5031, Eppendorf, Hamburg, Germany) at 45°C for 1 h. The extraction residues were dried for 2 d at room temperature for CT analyses. The dissolving of samples was conducted with 600 µL of water:methanol (1∶1, v/v) and analyzed by HPLC (Agilent 1100 Series HPLC Value System, Agilent Technologies, Santa Clara, California, US), with a diode array detector (DAD) and Zorbax RRHD SB-C18 column (2.1 mm×50 mm, 1.8 µm, Agilent Technologies). The injection volume for stem samples was 15 µL and for un-wounded leaf samples 20 µL and for wounded 10 µL, which was decided based on the first HPLC runs, indicating a high amount of tannin precursors in the wounded leaf samples. The identification of compounds was based on their retention times and spectral characteristics [60]. The commercial standard used in the quantification of compounds and the conductance of CT analyses can be found in Sutela *et al.* [40].

### Statistical analysis

The statistical examination of the data was carried out using the R 2.11.0 software [61] with the graphical user interface, the R Commander [62]. The growth and the lignin characteristics between clones and lines were examined using pairwise comparisons (the independent-samples t test, Welch Two Sample t test or Wilcoxon test). In the case of clone E5396 and line E5396/4 statistical testing was not conducted for lignin as the number of biological replicates was two. The Kruskal-Wallis test was used to test for differences in RGRs of larvae fed with among leaves of silver birch clones and lines. The beginning of autumn leaf senescence e.g. the yellowing of leaves between transgenic line (A1, A2 or A5) and non-transgenic clone A, was statistically examined with Pearson's Chi-squared test. The individual plants were initially classified to A) leaves are green; B) some yellowing leaves; C) less than 50% of leaves are yellowing; D) more than 50% of leaves are yellowing. To fulfil the requirements for Pearson's Chi-squared test the classes A and B and the classes C and D were combined when the data recorded at the 26^th^ of Sept. was tested. When the data recorded at the 2^nd^ of Oct. was tested the classes A and B and C were combined.

The relative expression of *Bp4CL* genes was examined by pairwise comparisons: the independent-samples t test, Welch Two Sample t test or Wilcoxon test in the case of abnormally distributed data. The Bonferroni correction was utilized for the pairwise tested relative expression differences of clones. The number of biological replicates was between 11–16 in the leaf samples and 3–5 in the stem samples within clone/line.

The differences in phenolic compounds and CTs of leaves were examined with the one-way Anova and Tukey Contrasts for the A, E5382, E5396 and R clones and lines A1, A5, and E5396/4 (n = 11). Square root and log(x+1) transformations were conducted to some of the variables. In addition, if the variable did not meet the assumptions required for Anova, the testing was conducted first with the Kruskal-Wallis followed with the two-sample t test, Welch Two Sample t test or Wilcoxon rank sum test. Statistical testing of line E5382/3 with lower number of biological replicates (n = 8) was conducted with the two-sample t test, Welch Two Sample t test or Wilcoxon rank sum test. The statistical analyzes of stem phenolics and CTs (n = 5) were the same as used for the leaf samples, however, in addition to line E5382/3, also the clone E5396 was examined with the two-sample t test, Welch Two Sample t test or Wilcoxon rank sum test because of the low number of biological replicates (n = 3). The Bonferroni correction was utilized for the *P*-values of pairwise tested variables in case of clones. The statistical testing of wounding effect within clone/line between the intact and wounded leaves collected 21 d after the treatment was conducted with the two-sample t test, Welch Two Sample t test or Wilcoxon rank sum test (n = 3–8).

## Results

### Characteristics of transgenic lines

The Southern blot analysis confirmed the *Pt4CL1* integration to silver birch genotypes A and E5382 (S5 Figure in S1 File). Only the presence of *nptII* was confirmed in the line E5396/4 with Southern blot (S6 Figure in S1 File) and the absence of the *Pt4CL1* expression was confirmed with real time RT-PCR (S3 and S4 Tables in S3 File), and thus the E5396/4 is called nptII line from now on. The copy numbers of Pt4CL1a constructs were 5, 1, and 1 in lines A1, A2, and A5 originated from clone A, respectively. The line E5382/3 generated from silver birch clone E5382 contained two copies of the Pt4CL1a constructs (S5 Figure in S1 File). Based on Northern blots, the *Pt4CL1* was expressed in the leaves, phloem and developing xylem of A1, A5 and E5382/3 lines (S7 Figure in S1 File). In the A2 line only the *nptII* was found to be expressed (S7 Figure in S1 File).

The transgenic lines A1, A2 and A5 were phenotypically similar to clone A, however the stem height was greater (*P*<0.05) in clone A than lines A1 and A2 throughout the three growing seasons (S6 Table in S4 File). Similarly, at the end of the second growing season, the stem diameter of clone A was wider (*P*<0.05) than in the lines A1 and A2, though no significant differences were detected in the stem diameter at the end of the third growing season. The shape of leaf margins was different in the first vegetative leaves of Pt4CL1a line E5382/3, however this phenotype was found to be transient (S8 Figure in S1 File). Moreover, the elongation growth of E5382/3 line was greater (*P*<0.05) at the end of both the first and second growing seasons. The growth of nptII line E5396/4 was altered significantly (*P*<0.05): the stems were drastically shorter and the stem diameters smaller in comparison with the clone E5396 (S6 Table in S4 File).

The autumn leaf senescence was followed during the end of the first growing season by monitoring the colour change of leaves. The leaf senescence proceeded similarly in clone E5382 and Pt4CL1a line E5382/3, whereas in the transgenic lines A1, A2 and A5 the senescence was delayed in comparison with clone A (S7 Table in S4 File, S9 Figure in S1 File). At first day monitored (the 26^th^ of September) 29 plant individuals of 51 in total were green or had few yellowing leaves in clone A. The number of plants with green leaves or few yellowing leaves were 49, 51 and 42 in clones A1 (χ^2^ = 21.8, df = 1, *P* = 3.03×10^−6^), A2 (χ^2^ = 28.1, df = 1, *P* = 1.18×10^−7^) and A5 (χ^2^ = 7.8, df = 1, *P* = 0.005), respectively. A week later, 43 plants of clone A had more than 50% of leaves yellowing whereas 15, 1 and 27 plants of lines A1 (χ^2^ = 31.3, df = 1, *P* = 2.17×10^−8^), A2 (χ^2^ = 70.5, df = 1, *P* = 2.2×10^−16^) and A5 (χ^2^ = 11.7, df = 1, *P* = 0.0006), respectively, were categorized to the same class. The Klason lignin contents of 2-year-old stems were similar among all clones and lines (Table 1). The S/G ratios of lines A1 and A5 were increased, but the difference was significant (*P*<0.05) only between the Pt4CL1a line A1 and clone A. No differences were detected in the RGRs of larvae in no-choice test among clones A and E5382 and lines A1, A2, A5, and E5382/3 (S8 Table in S4 File).

**Table 1:** The lignin analysis of 2-year-old silver birch stems.

Clone/Line	S/G	Acid soluble lignin %	Klason lignin %	Total lignin %
A clone	3.22±0.47	3.54±0.26	17.22±0.34	20.77±0.51
A1 line	3.97±0.06*	3.83±0.40	17.42±0.54	21.25±0.55
A2 line	2.95±0.19	3.27±0.36	18.21±0.19	21.49±0.39
A5 line	3.51±0.20	3.96±0.76	18.11±1.12	22.08±1.37
E5382 clone	3.61±0.12	3.93±0.54	17.96±0.96	21.89±1.14
E5382/3 line	3.50±0.31	4.00±0.35	18.41±1.13	22.41±0.83
E5396 clone	NA	3.95±0.11	17.1±0.23	22.22±0.39
E5396/4 line	NA	3.74±0.00	14.78±0.96	19.47±1.02

Values represent means ± SD. Star indicates significant differences (*P*<0.05) between clone and line according to the independent-samples t test, Welch Two Sample t test or Wilcoxon test. Number of replicates 4 expect for the E5396 and E5396/4 which had two biological replicates.

### Silver birch putative 4CLs

**Figure 1:**
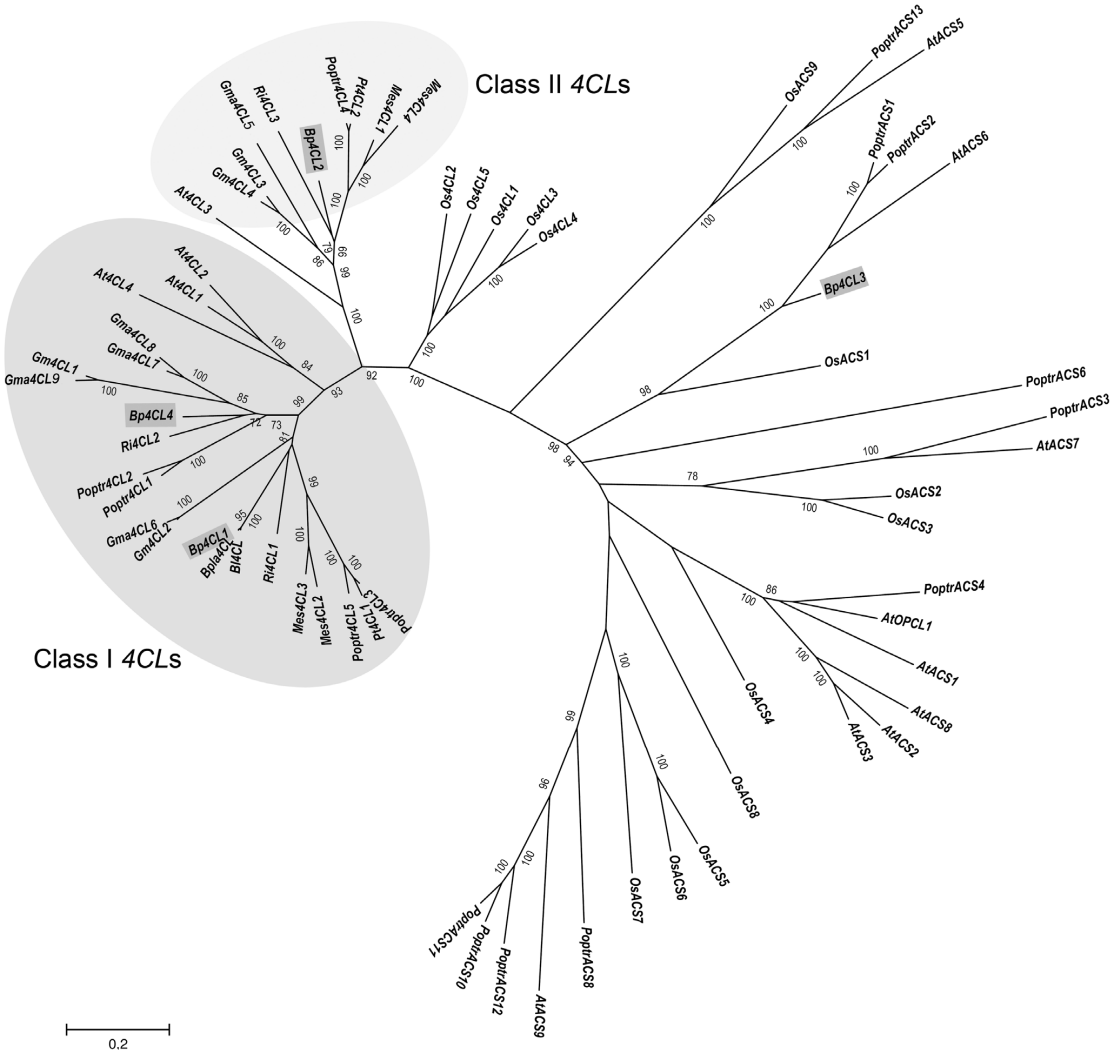
Phylogenetic analysis of silver birch (*Betula pendula*) *4CL* and *4CL*-like sequences. ML tree was constructed using the cds of silver birch putative *4CL* (*Bp4CL1*, *Bp4CL2*, *Bp4CL4*) genes and *4CL*-like (*Bp4CL3*) gene.

Four putative *4CL*-like mRNA sequences, *Bp4CL1-4*, were obtained from clone A and submitted to the GenBank with accession numbers KM099195-8. The alignments of predicted amino acid sequences of *Bp4CL1-4* are shown in S10-S13 Figures in S1 File. The 1629 bp long cds of *Bp4CL1* was 99% similar to the *4CL* sequences of *B. platyphylla* (AY792353) and *B. luminifera* (FJ410448). Furthermore, the predicted amino acid sequence of Bp4CL1 was identical with the translated 4CL sequence of *B. platyphylla*. At nucleotide level the 1725 bp long cds of *Bp4CL2* showed highest similarity (81%) with the predicted *4CL* mRNA of *Malus x domestica* (XM_008378682). At amino acid level the 4CL sequence of *Theobroma cacao* (XP_007029575) showed highest identity (84%) with predicted amino acid sequence of *Bp4CL1*. The cds of *Bp4CL3*, 1638 bp in length, showed 76% identity with *4CL*-like sequence of *Malus x domestica* (XM_008386493) at nucleotide level and 79% similarity with the AMP dependent CoA ligase of *Ricinus communis* (XP_002523698) at amino acid level. The 1629 bp long cds of *Bp4CL4* showed 79% similarity with *Eriobotrya japonica 4CL* sequence (KF767458) and 85% similarity at amino acid level with the *Fragaria vesca* subsp. *vesca* 4CL-like sequence (XP_004309949). The phylogenetic analysis was conducted for the classification of *4CLs* using a partial set of *4CL*-like *ACS* sequences presented by de Azevedo Souza *et al.* [23] supplemented with cds of *M*. *esculenta*, *G*. *max*, *R*. *communis*, *R*. *idaeus*, *P*. *tremuloides, B*. *platyphylla* and *B*. *luminifera 4CLs* (S5 Table in S3 File). The phylogenetic analysis suggested that *Bp4CL1* and *Bp4CL4* would belong to class I and *Bp4CL2* to class II *4CLs* (Fig. 1). The fourth *4CL* sequence, *Bp4CL3*, would belong to the group of *4CL*-like *ACS*.

The *Pt4CL1* gene was clustered to group of class I *4CL*s and showed greatest similarity with the *P*. *trichocarpa* Poptr4CL3. Of *Bp4CL* genes, the *Pt4CL1* showed highest identity with the *Bp4CL1*, 73% and 75% at nucleotide and amino acid level, respectively. The similarity of *Bp4CL2*, *Bp4CL3* and *Bp4CL4* were 61% and 55%, 60% and 56%, 40% and 59% at nucleotide and amino acid level with the cds and predicted amino acid sequence of *Pt4CL1*, respectively.

### Expression of silver birch 4CLs and Pt4CL1

In general, the expression of the putative *Bp4CL* genes was clone and line dependent and similar trends were present in the stems and leaves (Tables 2 and 3). However, in leaf samples, the variation was substantial within lines and clones which probably reflects differences in the developmental phase of individual leaves.

**Table 2:** The relative expression of *Bp4CL1 and Bp4CL2* genes in silver birch (*Betula pendula*) stems and leaves.

Gene		Stems				Leaves			
	Clone/Line	*Atub*		*PP2A*		*Atub*		*PP2A*	
***Bp4CL1***	A	1.27±0.21	ad	1.28±0.24	a	1.77±1.17	a	1.91±1.45	a
	A1	0.85±0.25	*	0.48±0.15	***	1.27±0.94		0.94±0.66	*
	A5	1.28±0.18		0.78±0.4	*	1.79±1.29		0.90±0.69	*
	E5382	0.52±0.05	bc	0.38±0.22	b	1.92±0.91	ab	0.63±0.40	b
	E5382/3	0.49±0.15		0.25±0.15		0.92±0.49		0.96±0.34	
	E5396	0.63±0.05	b	0.31±0.06	b	1.79±1.93	b	0.67±0.54	b
	E5396/4	0.82±0.15	*	0.31±0.11		1.48±1.14		0.64±0.38	
	R	1.82±0.15	cd	0.56±0.2	b	1.75±1.54	ab	1.14±0.45	ab
***Bp4CL2***	A	0.88±0.22	a	0.88±0.16	a	2.92±2.85		3.12±3.38	a
	A1	0.85±0.09		0.48±0.08	***	2.25±2.37		2.41±2.18	
	A5	1.17±0.54		0.71±0.19		4.22±3.87		2.70±2.17	
	E5382	0.40±0.25	ab	0.33±0.28	b	2.34±1.78		0.55±0.29	b
	E5382/3	0.13±0.09		0.06±0.04		0.50±0.31		0.51±0.14	
	E5396	0.29±0.09	b	0.14±0.05	b	1.50±1.39		0.33±0.10	b
	E5396/4	1.05±0.57	**	0.37±0.16	*	1.64±1.61		0.72±0.61	
	R	1.75±1.54	ab	1.14±0.45	ab	4.09±3.2		3.82±2.75	a

Values represent means and standard deviations calculated using *Atub* or *PP2A* gene as reference gene (n = 3–16). Statistical examination of data was performed using the independent-samples t test, Welch Two Sample t test or Wilcoxon test (with Bonferroni correction in the case of clonal differences). Different letters denote significant (*P*<0.05) difference between the clones. Stars denote significant (*P<0.05, **P<0.01, ***P<0.001) difference between transgenic line to non-transgenic clone.

**Table 3:** The relative expression of *Bp4CL3 and Bp4CL4* genes in silver birch (*Betula pendula*) stems and leaves.

Gene		Stems				Leaves			
	Clone/Line	*Atub*		*PP2A*		*Atub*		*PP2A*	
***Bp4CL3***	A	1.27±0.21	ad	1.28±0.24	a	0.30±0.61	a	0.29±0.67	a
	A1	0.85±0.25	*	0.48±0.15	***	0.38±0.88		0.31±0.60	
	A5	1.28±0.18		0.78±0.4	*	0	*	0	*
	E5382	0.52±0.05	bc	0.38±0.22	b	4.88±4.31	bc	0.79±0.24	b
	E5382/3	0.49±0.15		0.25±0.15		1.29±1.88	**	0.97±1.17	
	E5396	0.63±0.05	b	0.31±0.06	b	3.10±2.65	bc	0.91±0.70	b
	E5396/4	0.82±0.15	*	0.31±0.11		3.82±2.49		1.97±0.77	**
	R	1.82±0.15	cd	0.56±0.2	b	1.75±1.02	bc	2.30±1.59	c
***Bp4CL4***	A	0.88±0.22	a	0.88±0.16	a	5.15±3.83	a	6.01±4.00	a
	A1	0.85±0.09		0.48±0.08	***	3.36±2.79	*	2.40±1.44	**
	A5	1.17±0.54		0.71±0.19		2.84±2.40	*	1.40±1.05	***
	E5382	0.4±0.25	ab	0.33±0.28	b	0.43±0.40	b	0.15±0.31	b
	E5382/3	0.13±0.09		0.06±0.04		1.68±0.90	***	1.92±1.09	***
	E5396	0.29±0.09	b	0.14±0.05	b	0.63±0.82	bc	0.23±0.38	b
	E5396/4	1.05±0.57	**	0.37±0.16	*	1.81±1.53	*	0.93±0.51	***
	R	1.75±1.54	ab	1.14±0.45	ab	1.46±0.80	c	1.24±0.88	c

Values represent means and standard deviations calculated using *Atub* or *PP2A* gene as reference gene (n = 3–16). Statistical examination of data was performed using the independent-samples t test, Welch Two Sample t test or Wilcoxon test (with Bonferroni correction in the case of clonal differences). Different letters denote significant (*P*<0.05) difference between the clones. Stars denote significant (*P<0.05, **P<0.01, ***P<0.001) difference between transgenic line to non-transgenic clone.

In stems the *Bp4CL1* and *Bp4CL2* transcript levels were highest in the clone A, and significantly lower (*P*<0.05) in the clones E5382 and E5396 (Table 2). Likewise, the expression of *Bp4CL1* was significantly lower in clone R in comparison with the clone A. The transcript levels of *Bp4CL4* varied considerably within A lines/clone and R clone (Table 3). The *Bp4CL3* expression was significantly lower (*P*<0.05) in the clone A than clones E5382, E5396 and R. The expression of all *Bp4CL* genes were similar in clones E5382 and E5396. In the intact leaves the trends in the *Bp4CL1*-*4* transcript levels were somewhat similar to what found in stems (Tables 2 and 3).

The expression of *Bp4CL1*-*3* genes was at lower level in stems of the Pt4CL1a lines A1 and A5 in comparison with clone A (Tables 2 and 3). The stem transcript levels of *Bp4CL3* and *Bp4CL4* differed significantly between clone E5382 and Pt4CL1a line E5382/3. However, similar differences were also found between clone E5396 and nptII line E5396/4. The expression of *Bp4CL1* was found to be significantly altered in the stems of line A1 with both utilized reference genes (Tables 2 and 3). The leaf transcript levels of E5382 and E5382/3 and E5396 and E5396/4 resembled those ones found in stem samples. The *Pt4CL1* expressed both in the stems and leaves of Pt4CL1a lines A1, A5 and E5382/3 and showed highest levels in A1 line (S14 Figure in S1 File).

### Wounding induced the transcript levels of Bp4CL1 and Bp4CL2

**Figure 2:**
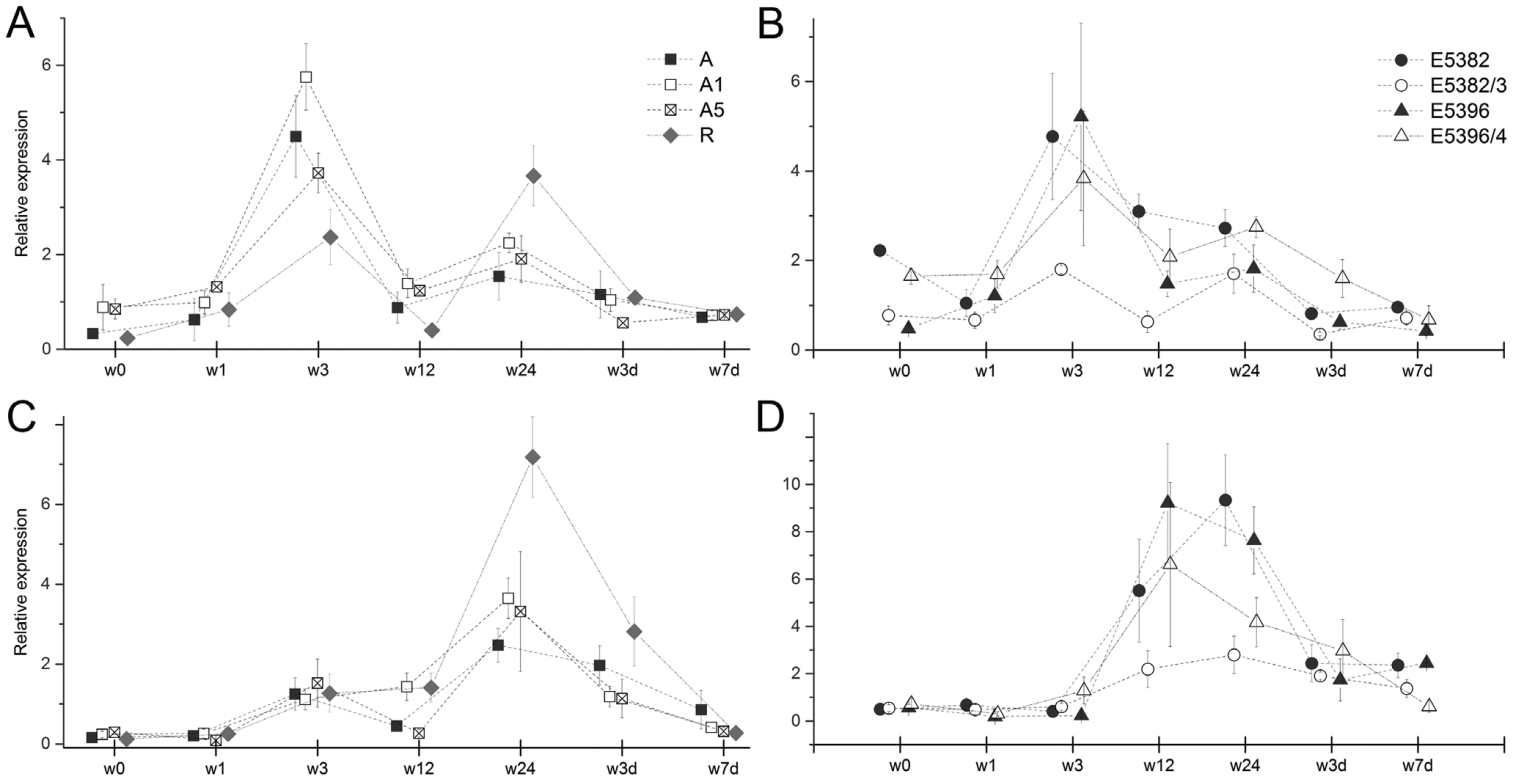
Relative expression of *Bp4CL1*-*4* genes in mechanically wounded silver birch (*Betula pendula*) leaves. The *Bp4CL1* (A, B) and *Bp4CL2* (C, D) expression in leaves collected immediately (w0) and 1, 3, 12, 24, 48, and 162 h after mechanical wounding of clones A and R and transgenic lines A1 and A5 (A, C) and clones E5382 and E5396 and transgenic lines E5382 and E5396 (B, D). Values represent mean ± SE calculated using the mean of control leaves as normalizer within each line/clone and *PP2A* as reference.

The *Bp4CL1* expression peaked 3 h after wounding in leaves of all studied clones and lines (Fig. 2A, B, S15 Figure in S1 File). After 24 h, the clone R showed clear induction in the *Bp4CL1* transcript levels, whereas the levels were somewhat increased in other clones and lines. In clones R and A, as well as in lines A1 and A5, the expression of *Bp4CL2* was highest in samples collected 24 h after the wounding (Fig. 2C, S15 Figure in S1 File). In clones E5382 and E5396 and in line E5396/4 the relative expression of *Bp4CL2* peaked at 12 h after wounding and in the case of clones E5396 and E5382 it stayed at higher level also 24 h after treatment (Fig. 2D). The *Bp4CL3* and *Bp4CL4* transcript levels did not respond to the wounding (S16 Figure in S1 File).

### Soluble phenolics and CTs of silver birches

**Figure 3:**
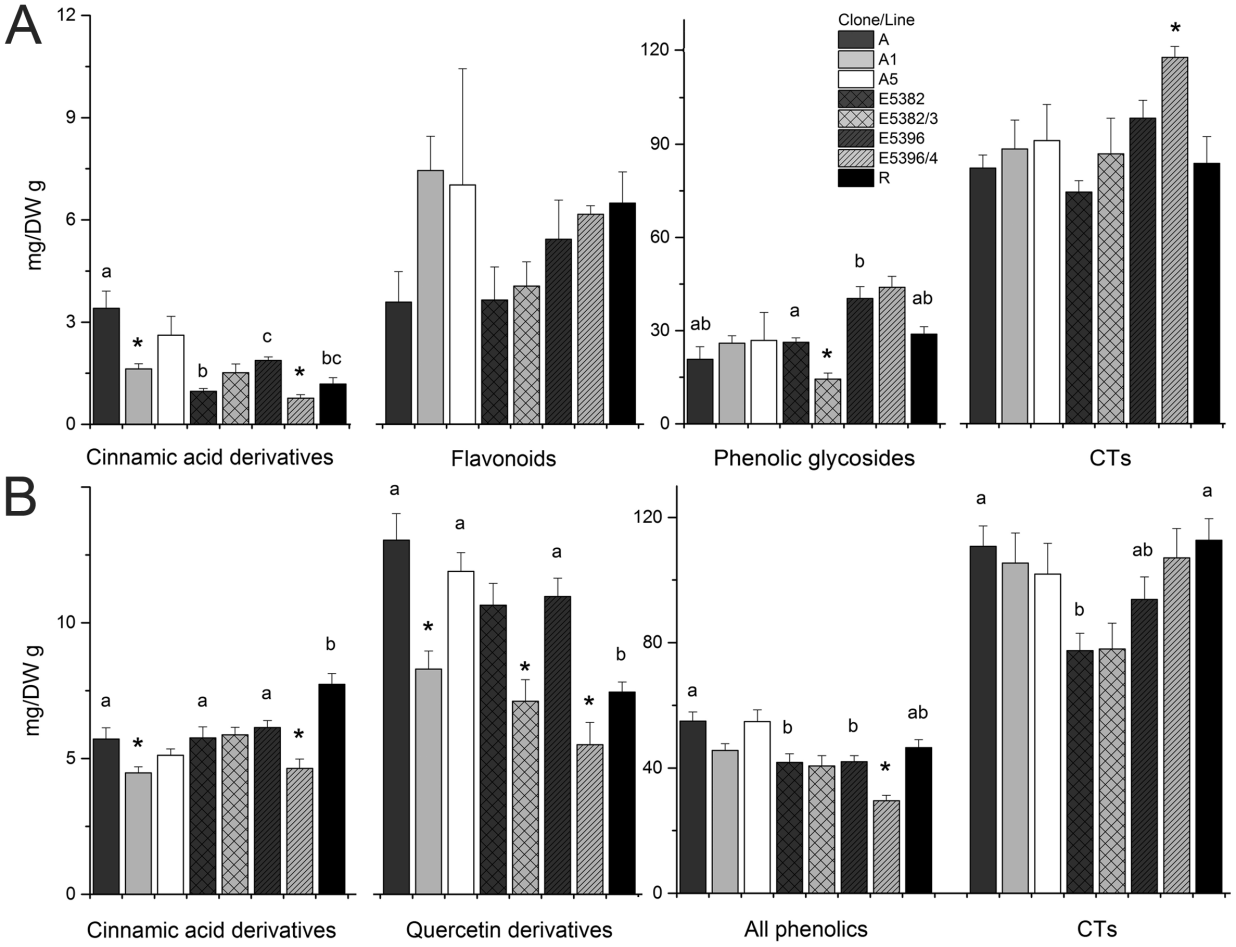
The contents of phenolic compounds and condensed tannins in silver birch (*Betula pendula*) stems and leaves. Stems (A) and leaves (B) of clones A, E5382, E5396, and R, and Pt4CL1a lines A1, A5 and E5282/3 and nptII line E5396/4. Values are mg/DW g mean ± SE. Different letters above the columns denote significant (*P*<0.05) difference between the clones. Stars above the columns of transgenic lines denote significant (*P*<0.05) difference between transgenic line to non-transgenic clone.

The cinnamic acid derivative content of clone A stems was prominent (Fig. 3A, S9 Table in S5 File). Two cinnamic acid derivatives were detected as trace amounts in clone A, but instead the concentration of *p*-OH-cinnamic acid glucoside was at highest level, resulting in significantly (*P*<0.05) higher total content of cinnamic acids. In stems of clone E5396, the content of *p*-OH-cinnamic acid glucoside was significantly (*P*<0.05) higher than in clones E5382 and R, and also a significant difference was found between clones E5382 and E5396 in the content of total cinnamic acid derivatives. Of individual flavonoids the concentration of a myricetin derivative was significantly (*P*<0.05) lower in clone A than clone R, however, the total flavonoid concentrations were similar in all studied clones (Fig. 3A). The total phenolic glycoside content was greatest in clone E5396 caused by the high platyfylloside content, however, the differences were significant only between clones E5396 and E5382. The salidroside content of stems was significantly lower in clone A than in the clones E5382 and R (S9 Table in S5 File).

The concentration of catechin derivative, 3,4′-dihydroxypropiophenone 3-glucoside, and *p*-OH-cinnamic acid glucoside (Fig. 3A, S9 Table in S5 File) in stems differed significantly from clone A and one or both of the Pt4CL1a lines A1 and A5. The phenolic concentrations of stems differed most between clone E5382 and line E5382/3. Of individual compounds concentrations of myricetin derivative, *p*-OH-cinnamic acid glucoside, *p*-OH-cinnamic acid derivative, platyfylloside, salidroside and catechin derivative differed significantly and were lower in the E5382/3 than in the non-transgenic clone apart of catechin derivative (S9 Table in S5 File). When the total contents were compared, phenolic glycosides as well as the concentration of all phenolic compounds were found to be significantly reduced in the line E5382/3 (Fig. 3A, S9 Table in S5 File). Between clone E5396 and nptII line E5396/4 significant variation was found in the concentrations of cinnamic acids, apigenin derivative, salidroside, and soluble CTs (Fig. 3A, S9 Table in S5 File).

In general, greater variation was found in the phenolic compounds among clones in leaves than in stems. Clones A and R showed specific phenolic profiles, whereas the phenolic contents of clones E5382 and E5396 were more similar (Fig. 3B, S10 Table in S5 File). The cinnamic acid and *p*-OH-cinnamic acid derivatives were both significantly (*P*<0.05) higher in clone R than in other clones. In addition, the concentrations of kaempferol and myricetin 3-rhamnosides and apigenin derivatives were significantly (*P*<0.05) higher in clone R. Kaempferol, myricetin and quercetin 3-acetyl-glucosides, as well as quercetin derivative, were detected in clone A but found absent or at very low concentrations in clones E5382, E5396 and R. In addition, the leaves of clone A contained myricetin 3-arabinose not detected in other clones. The total flavonoid concentration as well as the total content of HPLC identified phenolic compounds was higher (*P*<0.05) in clone A. Moreover, the concentration of soluble CTs and total CTs was highest in clone A (Fig. 3B, S10 Table in S5 File).

The leaf contents of cinnamic acid and quercetin derivatives as well as insoluble CTs varied significantly (*P*<0.05) between clone A and Pt4CL1a line A1 (Fig. 3B, S10 Table in S5 File). One cinnamic acid derivative was significantly higher in line A5. Moreover, significant difference between clone A and line A5 was found in the concentration of neolignan which was, in addition, significantly reduced in A1 line. Between clone E5382 and Pt4CL1a line E5382/3, as well as between clone E5396 and line E5396/4, significant differences were detected in the concentration of chlorogenic acid, cinnamic acid derivatives, hyperin and quercetin derivatives (Fig. 3B, S10 Table in S5 File).

### Wounding caused only some changes in the phenolic compound concentrations

**Figure 4:**
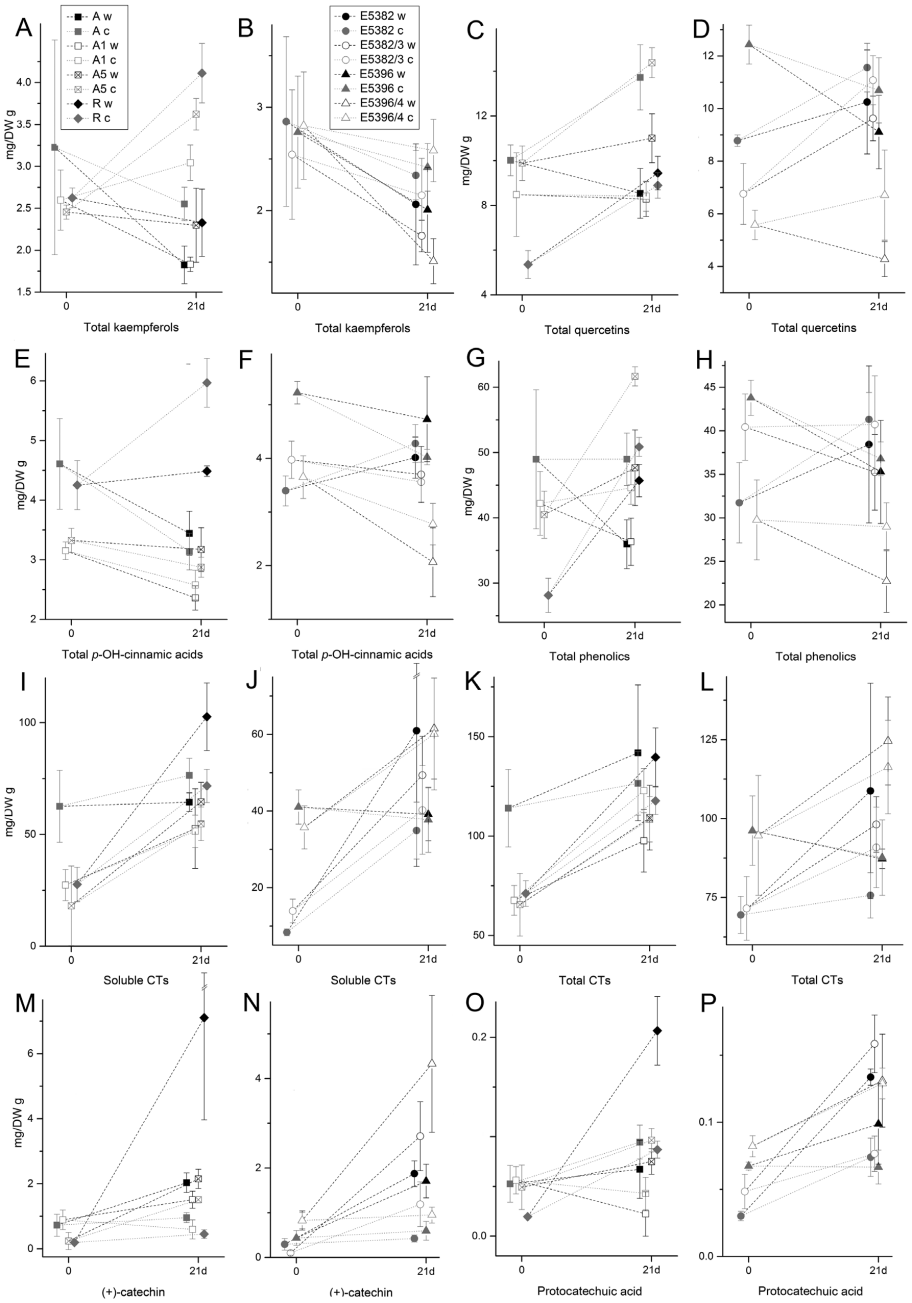
Wounding effects on the leaf phenolics of silver birch (*Betula pendula*). The contents of kaempferols (A, B), quercetin (C, D), and *p*-OH-cinnamic acid derivatives (E, F), total content of HPLC identified phenolics (G, H), soluble CT (I, J), total CT (K, L), (+)-catechin (M, N) and protocatechuic acid (O, P) concentrations in untreated (c) and mechanically wounded (w) leaves collected at day 0 and 21 d after treatment in clones A and R and Pt4CL1a lines A1 and A5 (A, C, E, G) and clones E5382 and E5396 and transgenic lines E5382/3 and E5396/4 (B, D, F, H). Values are mg/DW g mean ± SE.

The effect of mechanical wounding to phenolic compounds was studied with leaf samples collected 21 days after treatment. In general, the wounding caused reduction in the quercetin, kaempferol as well as total flavonoid concentrations (Fig. 4, S11 Table in S5 File). No apparent trend was detected in the content of cinnamic acid derivatives, whereas concentrations of soluble CTs increased in all lines and clones apart of clone A (Fig. 4, S11 Table in S5 File).

Otherwise, the levels of phenolic compounds altered more due to the wounding in clones A and R than in clones E5382 and E5396, as only few significant changes were detected in the concentration of individual compounds (S11 Table in S5 File). Of clones A and R the latter had greater response found in particular in the increased concentration of (+)-catechin, neolignan, protocatechuic acid, and soluble CTs (Fig. 4). The clones and corresponding transgenic lines responded similarly to the wounding.

## Discussion

The 4CL isoforms are able to use a variety of hydroxycinnamic acids as substrates, and hence, the extent of gene family expansion and substrate diversity has been found to be connected [8]. In various plant species, the biosynthesis of monolignols has been considered to be the main function of the class I *4CL* genes [9], [14]–[20]. The class II *4CL* genes have been suggested to function, in addition to lignin biosynthesis, in the synthesis of flavonoids [9], [17]–[19], [63]. In the present study, four putative silver birch *4CL* genes were cloned and their expression was monitored in stems and leaves of silver birches. The phylogenetic ML tree, reconstructed using cds of characterized *4CL* genes and *4CL*-like *ACS* genes, was in accordance with the general view of class I and II members [13], [18], [64] as well as with the study of de Azevedo Souza *et al.* [23] presenting the evolution of *4CL*-like *ACS* genes based on their translated nucleotide sequences. The phylogenetic analysis suggested that two of the putative *4CL* genes of silver birch belong to class I and one *4CL* gene would belong to class II. The three *4CL* genes, *Bp4CL1*, *Bp4CL2*, and *Bp4CL4*, were expressed in stems of all studied silver birch clones.

The *4CL*-like *ACS*s are a land plant-specific group of genes encoding adenylate-forming enzymes which contain C-terminal consensus PTS1 peroxisomal target sequence [23]. The *4CL*-like *ACS* genes of *P*. *trichocarpa* (*PoptrACS1*, *PoptrACS2*), Arabidopsis (*AtACS6*) and rice (*OsACS1*) were shown to form one of the five conserved clades of *4CL*-like *ACS* sequences and were detected to express in various organs and tissues of Arabidopsis and *P*. *trichocarpa* [23]. In the present study, the phylogenetic analyses suggested that *Bp4CL3* would be *4CL*-like *ACS* gene and furthermore, would belong to the same clade with *PoptrACS1*, *PoptrACS2*, *AtACS6* and *OsACS1*. The *Bp4CL3* expression levels varied considerably among studied silver birch clones being, however, below detection level with used qPCR method in the leaves and stems of clone A. The *Bp4CL3* showed no response to mechanical wounding. Similarly, the *AtACS6* and *PoptrACS2* were shown to be unresponsive to mechanical wounding as well as to simulated herbivory and herbivory treatments [23]. However, wounding and herbivory induced the expression of *PoptrACS1* and *4CL*-like *ACS* genes of other clades indicating that some *4CL*-like *ACS* genes also may function in defence processes [23].

The antisense strategy with *4CL* genes has been successfully utilized with several plant species to reduce the lignin content [16], [20], [25], [28], [29]. In the present study, silver birch elite clones A, E5382 and E5396 were transformed with the pRT99/35S-*Pt4CL1*-a and five silver birch lines were subsequently regenerated. Of these A1, A5 and E5382/3 originating from clones A and E5382, respectively, were confirmed to express *Pt4CL1* under standard greenhouse conditions. The Pt4CL1a lines were shorter than the non-transgenic clones during the first growing season, however, at the end of third growing season, the heights differed only between clone A and Pt4CL1a line A1. Moreover, the growth of transgenic silver birch lines only expressing *nptII* (A2, E5396/4) was altered, possibly reflecting the unfavourable insertion site of transgene/s. Furthermore, the autumn leaf senescence, followed during the end of first growing season, was delayed especially in the A2 line but also in some extent in lines A1 and A5 in comparison with the clone A. The delay on the leaf senescence was probably connected to the differences in plant heights as the shortest plants turned the leaf colour last and hence, could partly reflect shaded light conditions [65]. The leaves of second growing season were of similar food quality for defoliators among clones and lines indicating that the transformation did not cause drastic alterations in the leaf chemistry of silver birch lines.

The *Pt4CL1* in antisense orientation did not cause changes in the lignin content of silver birch lines and moreover, altered lignin composition was detected only in the Pt4CL1a line A1. The relative expression of *Pt4CL1* was greatest in A1 line which, besides, showed highest copy number of integrated Pt4CL1a constructs. Voelker et al. [29] demonstrated with hybrid poplar that lignin quantity and S/G ratio can be modified by reducing the *4CL1-1* (homolog of *Poptr4CL3*) levels around 60% and the *4CL2-2* (*Poptr4CL5* homolog) levels around 95% of the expression levels in control poplar. In the present study, the *Pt4CL1* shared greatest similarity with the cds sequence of *Bp4CL1* and, indeed, significant alteration was found in the *Bp4CL1* expression of Pt4CL1a A1 stems. However, the *Bp4CL1* levels of A1 line were still more than 60% of the control clone A when *Atub* was utilized as the reference gene, possible explaining why the lignin content of A1 line remained unchanged. Moreover, it is likely that in Pt4CL1a lines A5 and E5382/3, the expression levels of *Pt4CL1* were not sufficient to cause required reduction in the transcript levels of *Bp4CL1* to cause changes in the lignin composition.

Wounding has been shown to induce the expression of genes encoding enzymes of the general phenylpropanoid route leading to the generation of monolignols essential for the wound sealing [19]. Thus, the phenylpropanoid flux should be directed from the flavonoid biosynthesis route towards biosynthesis of monolignols and, indeed, wounding has been shown to decrease the expression of potential lignin biosynthesis repressors [66], [67]. In the present study, the expression of *Bp4CL1* and *Bp4CL2* was induced transiently 3 and 24 h after wounding, respectively. On the contrary, the class I gene, *Bp4CL4*, was unresponsive to wounding treatment. The induction of *4CL* genes has been detected by monitoring the transcript levels and/or by promoter analyses using potato (*Solanum tuberosum*) [14], Arabidopsis [9], [19], *P*. *tremuloides* [68], tea (*Camellia sinensis*) [69], rice [64], *Salvia miltiorrhiza* [70], and kernel (*Hibiscus cannabinus*) [71]. The expression and promoter studies have demonstrated that the regulation of *4CL*s is complex and includes elements, positioned both upstream and also within the cds, acting positively and negatively [19] and, thus different *4CL* family members show distinct expression patterns due to wounding [9], [19], [64]. In Arabidopsis, the class I *4CL* genes *At4CL1* and *At4CL2* showed biphasic induction with expression peaks 2.5 h and 48 h after wounding [19]. The response of the *At4CL3* connected to flavonoid biosynthesis was first reduced and then steadily increased up to the 72 h after wounding which represented the latest time point monitored [19]. When compared to the expression patterns of *Bp4CL1* and *Bp4CL2*, in the present study, they were similar with the early responses of *At4CL1*, *At4CL2* and *At4CL3* in Arabidopsis as described by Soltani *et al.* [19].

The genotype of silver birch has substantial influence on the phenolic composition [72]–[75]. In the present study, soluble phenolic compounds varied in all four studied silver birch clones and differences were detected in both stems and leaves in the level of individual compounds, as well as compound groups such as cinnamic acids and phenolic glycosides. In Pt4CL1a lines and clones significant differences were detected mostly in the concentrations of individual compounds, although, reduced concentration of cinnamic acid derivatives and phenolic glycosides were detected in individual Pt4CL1a lines. In addition, the E5396/4 line expressing only *nptII* showed altered phenolic compound concentrations in comparison with the non-transgenic clone E5396. This indicates that, in addition to the aberrant growth, the insertion site/s of transgenes may have caused alterations to the phenylpropanoid pathway which was, in addition, found as changed transcript levels of silver birch *4CL* genes. The decrease in the *4CL* expression has led to altered flux of intermediates of the phenylpropanoid route which has been shown to increase the content of cinnamic acid derivatives [11], [20], [25], [28] and other phenolics [29], [32]. In the present study, however, the Pt4CL1a line A1 having altered S/G ratio, showed only increase in the (+)-catechin content of stems and the insoluble CT content of leaves.

In general, the production of phenolic compounds is induced in woody deciduous plants upon damage, however, the response varies according to the studied species, plant ontogeny, growth rate, and timing of the damage [7], [76], [77]. In the present study, the wounding caused only minor changes in the studied soluble phenolic compounds examined of leaves collected 21 d after wounding. As an overall trend the derivatives of kaempferol and quercetin as well as total concentration of flavonoids were reduced in the injured leaves. Similarly Muilenburg *et al.* [78] found reduction in the content of low molecular weight phenolics in silver birch and *B*. *papyrifera* and proposed that phenolics were possibly utilized for the production of more complex polyphenols or polymers such as lignin. In the present study, the wounding response varied between clones: E5382 and E5396 responded mildly whereas clone R showed the strongest response, which was found, for instance, as the elevated concentrations of neolignan, protocatechuic acid and (+)-catechin, the latter of which can be utilized as a precursor for CT synthesis.

Wounding has been reported to increase the CT concentrations in *P*. *tremuloides* [79] and in turtlegrass (*Thalassia testudinum*) [80]. Moreover, MYB134, which regulates genes related to the biosynthesis of CTs, has been shown to be wound inducible [81]. However, the induction of CT synthesis upon herbivory is species specific [7]. For instance, defoliation of silver birch did not cause induction in the leaf CT levels [72] and wounding of silver birch and *B*. *papyrifera* stems caused reduction in CTs [78]. Similarly, leaf damage treatments of *Quercus* species and *Acer opalus* ssp. *granatense* [82]–[84] caused reduction in the tannin levels. In the present study, however, soluble CTs increased while insoluble CTs decreased in the leaves of clone R. This, together with increased (+)-catechin content, suggests that CT accumulation had possible not reached its maximum in clone R.

The 4CLs have been studied extensively because of their central role in the general phenylpropanoid and monolignol biosynthesis route leading to lignin with economic importance and a vital role for the proper function of wood. However, in the light of recent findings [85]–[87], it seems that the studies on the monolignol biosynthesis route are far from over. In general, the individual *4CL* genes have been considered to have plant part specific expression patterns related to developmental and non-developmental processes. However, the function and regulation of 4CLs has been found to be more complex, for instance, Chen *et al.* [87] showed that subunits of two *P*. *trichocarpa* 4CLs, 4CL3 and 4CL5, were able to interact and form heterotetrameric protein complex which affects the direction and rate of metabolite fluxes.

In the present study we show that silver birch has at least two class I and one class II *4CL* genes having clone specific expression patterns as well as one *4CL*-like *ACS* gene. The results indicate that class I *4CL*, *Bp4CL1*, functions in the monolignol biosynthesis route as the transcript levels were reduced in the Pt4CL1a line A1 having altered S/G ratio. The *Pt4CL1* did not cause drastic alterations in the phenolics of studied Pt4CL1a, lines perhaps caused by insufficient transcript levels and/or developmental stage of young plants. The specific functions of silver birch 4CLs remain to be solved. However, *Bp4CL1* could serve as a candidate to alter the lignin characteristics in silver birch.

## Supporting Information

S1 File **This file contains S1-S16 Figures. S1 Figure.** A schematic presentation of the pRT99/35S-Pt4CL1-a plasmid vector used in the biolistic transformation of silver birch clones A, E5382 and E5396. **S2 Figure.** The scatterplots presenting CV% and concentration values of stem samples. The CV% (SD_Cq_/mean_Cq_) values were calculated from two technical replicates of each utilized primer pair and the concentration values were generated with Abs Quant/2nd Derivative Max for All Samples Analysis of Lightcycler 480 Software release 1.5.0 SP3. A, *Atub*; B, *PP2A*; C, *Bp4CL1*; D, *Bp4CL2*; E, *Bp4CL3*; F, *Bp4CL4*; G, *Pt4CL1*. **S3 Figure.** The scatterplots presenting CV% and concentration values of leaf samples. The CV% (SD_Cq_/mean_Cq_) values were calculated from two technical replicates of each utilized primer pair and the concentration values were generated with Abs Quant/2nd Derivative Max for All Samples Analysis of Lightcycler 480 Software release 1.5.0 SP3. A, *Atub*; B, *PP2A*; C, *Bp4CL1*; D, *Bp4CL2*; E, *Bp4CL3*; F, *Bp4CL4*; G, *Pt4CL1*. **S4 Figure.** The Cq values of *Atub* and *PP2A* in undamaged and wounded leaves. The Cq values represent crossing point values generated with Abs Quant/2nd Derivative Max for All Samples Analysis of Lightcycler 480 Software release 1.5.0 SP3 of *Atub* (A) and *PP2A* (B) in non-treated leaves (c) and leaves collected immediately after mechanical wounding (w0) and 1 (w1h), 3 (w3h), 12 (w12h), 24 (w24h), 72 (w3d) and 168 (w7d) hours after the wounding treatment. **S5 Figure.** Southern blot analysis of regenerated silver birch lines. Lines A1, A2, A5 and E5382/3 were transformed with pRT99/35S*-Pt4CL1*-a plasmid vector and probed for the presence of the 35S*-Pt4CL1* and *nptII*. A genomic DNA sample of 15 µg digested with *Bam*HI (figures on the left) or *Xba*I (figures on the right) was loaded to each lane. **S6 Figure.** Southern blot analysis of regenerated silver birch line E5396/4. Line was transformed with pRT99/35S*-Pt4CL1*-a plasmid vector and probed for the presence of the *nptII*. A genomic DNA sample of 15 µg digested with *Bam*HI (figures on the left) or *Xba*I (figures on the right) was loaded to each lane. **S7 Figure.** Northern blot analysis of transgenic silver birch lines. RNA samples of 15 µg were isolated from the leaf (L), phloem (P) and xylem (X) of clones A and E5382 and lines A1, A2, A5 and E5382/3. The probes were 1.1 and 0.8 kb for Pt4CL1 and nptII, respectively. **S8 Figure.** The leaf morphology of Pt4CL1a line E5382/3. During the first growing season in the greenhouse, the leaf margins of Pt4CL1a line E5382/3 (on the left) differed in from clone E5382 (on the right). **S9 Figure.** The autumn leaf senescence of silver birch clones and lines. The clone A, line A2, line A5, line E5382/3, and clone E5382 (from left to right) at the end of first growing season at the greenhouse of Finnish Forest Research Institute Punkaharju Unit (photographed on the 6^th^ of October). **S10 Figure.** Alignment of predicted amino acid sequence of silver birch (*Betula pendula*) putative 4-coumarate:CoA ligase (Bp4CL1, KM099195). Bp4CL1 aligned with the predicted amino acid sequences of *Betula luminifera* (Bpl4CL1, AY792353), *Betula platyphylla* (Bl4CL, FJ410448), *Glycine max* (Gm4CL2, Glyma13g44950; Gm4CL6, Glyma15g00390), *Manihot esculenta* (Mes4CL2, cassava4.1_005014m; Mes4CL3, cassava4.1_005006m), *Populus tremuloides* (Pt4CL1, AF041049), and *Populus trichocarpa* (Poptr4CL3, grail3.0100002702 LG I; Poptr4CL5, fgenesh4_pg.C_LG_III001773 LG III). **S11 Figure.** Alignment of predicted amino acid sequence of silver birch (*Betula pendula*) putative 4-coumarate:CoA ligase (Bp4CL2, KM099196). Bp4CL2 aligned with the predicted amino acid sequences of *Arabidopsis thaliana* (At4CL3, At1g65060), *Rubus idaeus* (Ri4CL3, AAF91308), *Glycine max* (Gm4CL3, NM_001250341), *Manihot esculenta* (Mes4CL1, cassava4.1_004658m; Mes4CL4, cassava4.1_004136m), *Populus tremuloides* (Pt4CL2, AF041050), and *Populus trichocarpa* (Poptr4CL4, grail3.0099003002 LG IX). **S12 Figure.** Alignment of predicted amino acid sequence of silver birch (*Betula pendula*) putative 4CL-like acyl-CoA synthetase (ACS) (Bp4CL3, KM099197). Bp4CL3 aligned with the predicted amino acid sequences of *Arabidopsis thaliana* (AtACS6, At4g05160), *Oryza sativa* (OsACS1, Os03g05780) and *Populus trichocarpa* (PoptrACS1, eugene3.01230068; PoptrACS2, estEXT_fgenesh1_pg_v1.C_LG_IV0024). **S13 Figure.** Alignment of predicted amino acid sequence of silver birch (*Betula pendula*) putative 4-coumarate:CoA ligase (Bp4CL4, KM099198). Bp4CL4 with the predicted amino acid sequences of *Rubus idaeus* (Ri4CL2, AAF91309), *Glycine max* (Gm4CL7, Glyma17g07170; Gm4CL8, Glyma17g07180; Gm4CL1, Glyma17g07190; Gm4CL9, Glyma13g01080), and *Populus trichocarpa* (Poptr4CL1, estExt_fgenesh4_pg.C_1210004 scaffold3; Poptr4CL2, gw1.XVIII.2818.1 LG_XVIII). **S14 Figure.** Relative expression of *Pt4CL1* in stems and leaves of Pt4CL1a lines A1, A5 and E5282/3. Values represent means and standard deviations calculated using *PP2A* gene as reference gene (n = 3–14). **S15 Figure.** Relative expression of *Bp4CL1*-*4* genes in mechanically wounded silver birch (*Betula pendula*) leaves. The *Bp4CL1* (A, C) and *Bp4CL2* (B, D) expression in leaves collected immediately (w0) and 1 (w1), 3 (w3), 12 (w12), 24 (w24), 72 (w3d), and 162 (w7d) h after mechanical wounding of clones A, E5382, E5396 and R and transgenic lines A1, A5, E5382/3 and E5396/4. Values represent means and standard errors calculated from the target/refence ratios. A and C, *PP2A* was used as the reference gene; B and D, *Atub* was used as the reference gene. **S16 Figure.** Relative expression of *Bp4CL3* (A, B), and *Bp4CL4* (C, D) in wounded leaves. The leaves were collected immediately (w0) and 1, 3, 12, 24, 48, and 162 h after mechanical wounding of silver birch (*Betula pendula*) clones A and R and transgenic lines A1 and A5 (A, C) and clones E5382 and E5396 and transgenic lines E5382 and E5396 (B, D). Values represent means and standard errors calculated using the mean of control leaves as normalizer within each line/clone and *PP2A* as reference.
(PDF)

S2 File **This file contains S1-S2 Tables. S1 Table.** The primers used in the production the probes, subcloning of cds of putative *Bp4CL1-4* and real-time RT-PCR. **S2 Table.** The performance of *Atub* and *PP2A* primers used in the amplification of reference genes.
(PDF)

S3 File **This file contains S3–S5 Tables. S3 Table.** The Cq (crossing point) and concentration values of stems. **S4 Table.** The Cq (crossing point) and concentration values of leaves. **S5 Table.** The *4CL* and *4CL*-like *ACS* sequences used in the generation of ML tree.
(XLS)

S4 File **This file contains S6–S8 Tables. S6 Table.** The growth characteristics of silver birch non-transgenic clones and transgenic lines. **S7 Table.** The phenology of autumn leaf senescence at the end of first growing season. **S8 Table.** Relative growth rates (mg/d) of lepidopteran larvae. The leaves of silver birch clones (A and E5382) and lines (A1, A2, A5 and E5382/3) were offered to the larvae of *Aethalura punctulata*, *Cleora cinctaria*, *Epirrita autumnata*, and *Orthosia gothica*.
(PDF)

S5 File **This file contains S9–S11 Tables. S9 Table.** Concentrations of phenolic compounds in silver birch stems. **S10 Table.** Concentrations of phenolic compounds in silver birch leaves. **S11 Table.** Concentrations of phenolic compounds in silver birch leaves 21 d after wounding.
(XLS)
