# Supplementary material for: Phenolic Compounds and Expression of 4CL Genes in Silver Birch Clones and Pt4CL1a Lines
Source: PLoS One. 2014 Dec 11;9(12):e114434. doi: 10.1371/journal.pone.0114434 (PMC4263613; doi:10.1371/journal.pone.0114434)
Supplement: S1 File — This file contains S1-S16 Figures. S1 Figure. A schematic presentation of the pRT99/35S-Pt4CL1-a plasmid vector used in the biolistic transformation of silver birch clones A, E5382 and E5396. S2 Figure. The scatterplots presenting CV% and concentration values of stem samples. The CV% (SDCq/meanCq) values were calculated from two technical replicates of each utilized primer pair and the concentration values were generated with Abs Quant/2nd Derivative Max for All Samples Analysis of Lightcycler 480 Software release 1.5.0 SP3. A, Atub; B, PP2A; C, Bp4CL1; D, Bp4CL2; E, Bp4CL3; F, Bp4CL4; G, Pt4CL1. S3 Figure. The scatterplots presenting CV% and concentration values of leaf samples. The CV% (SDCq/meanCq) values were calculated from two technical replicates of each utilized primer pair and the concentration values were generated with Abs Quant/2nd Derivative Max for All Samples Analysis of Lightcycler 480 Software release 1.5.0 SP3. A, Atub; B, PP2A; C, Bp4CL1; D, Bp4CL2; E, Bp4CL3; F, Bp4CL4; G, Pt4CL1. S4 Figure. The Cq values of Atub and PP2A in undamaged and wounded leaves. The Cq values represent crossing point values generated with Abs Quant/2nd Derivative Max for All Samples Analysis of Lightcycler 480 Software release 1.5.0 SP3 of Atub (A) and PP2A (B) in non-treated leaves (c) and leaves collected immediately after mechanical wounding (w0) and 1 (w1h), 3 (w3h), 12 (w12h), 24 (w24h), 72 (w3d) and 168 (w7d) hours after the wounding treatment. S5 Figure. Southern blot analysis of regenerated silver birch lines. Lines A1, A2, A5 and E5382/3 were transformed with pRT99/35S-Pt4CL1-a plasmid vector and probed for the presence of the 35S-Pt4CL1 and nptII. A genomic DNA sample of 15 µg digested with BamHI (figures on the left) or XbaI (figures on the right) was loaded to each lane. S6 Figure. Southern blot analysis of regenerated silver birch line E5396/4. Line was transformed with pRT99/35S-Pt4CL1-a plasmid vector and probed for the presence of the nptII. A genomic DNA [file pone.0114434.s001.pdf]

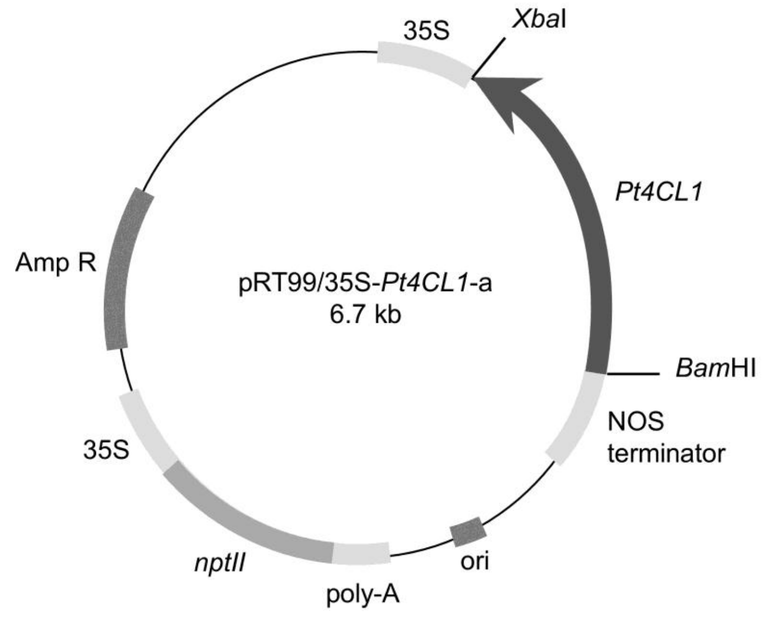

Figure S1. A schematic presentation of the pRT99/35S-Pt4CL1-a plasmid vector used in the biolistic transformation of silver birch clones A, E5382 and E5396.

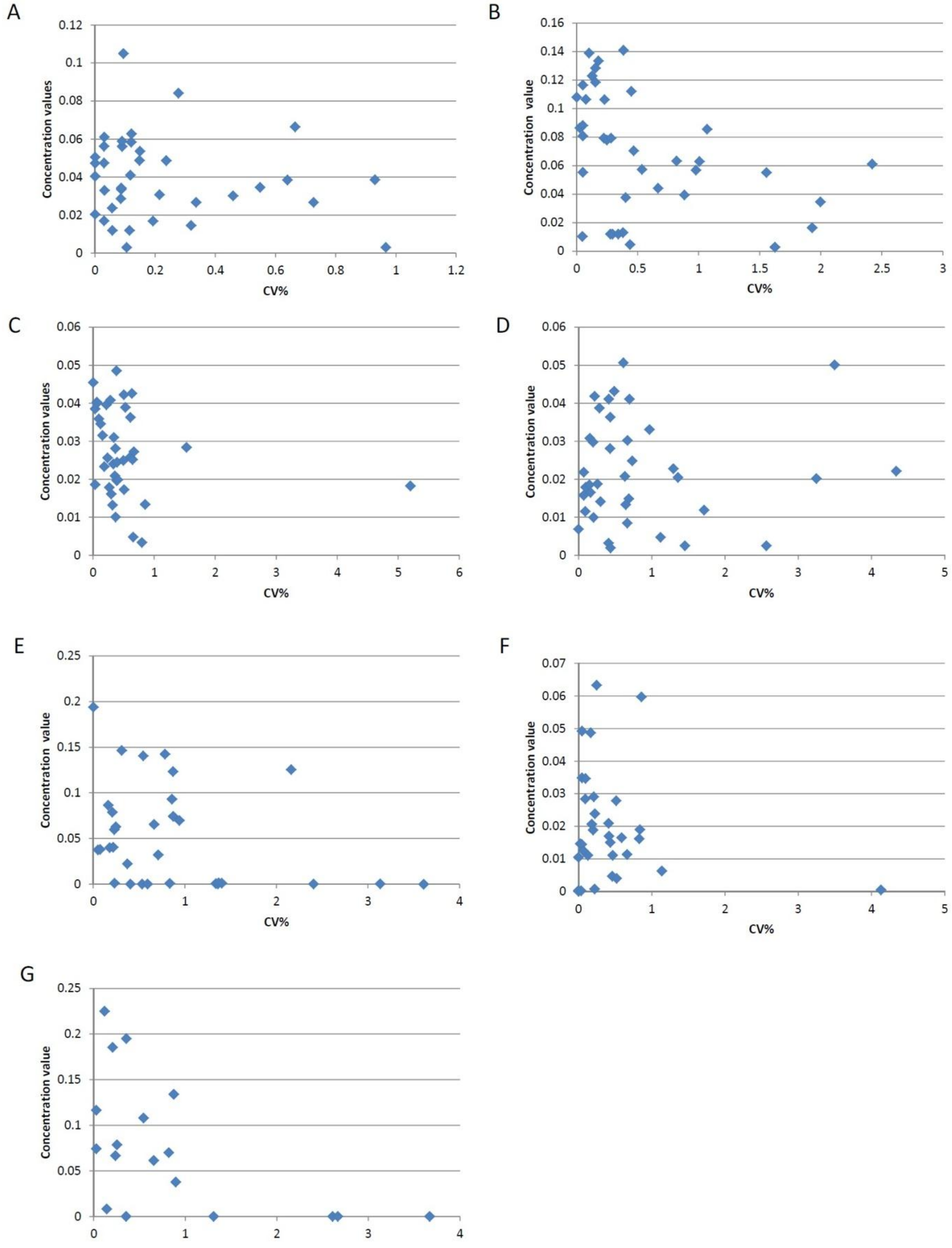

Figure S2. The scatterplots presenting CV% and concentration values of stem samples. The CV% ($SD_{Cq}/mean_{Cq}$) values were calculated from two technical replicates of each utilized primer pair and the concentration values were generated with Abs Quant/2nd Derivative Max for All Samples Analysis of Lightcycler 480 Software release 1.5.0 SP3. A, *Atub*; B, *PP2A*; C, *Bp4CL1*; D, *Bp4CL2*; E, *Bp4CL3*; F, *Bp4CL4*; G, *Pt4CL1*.

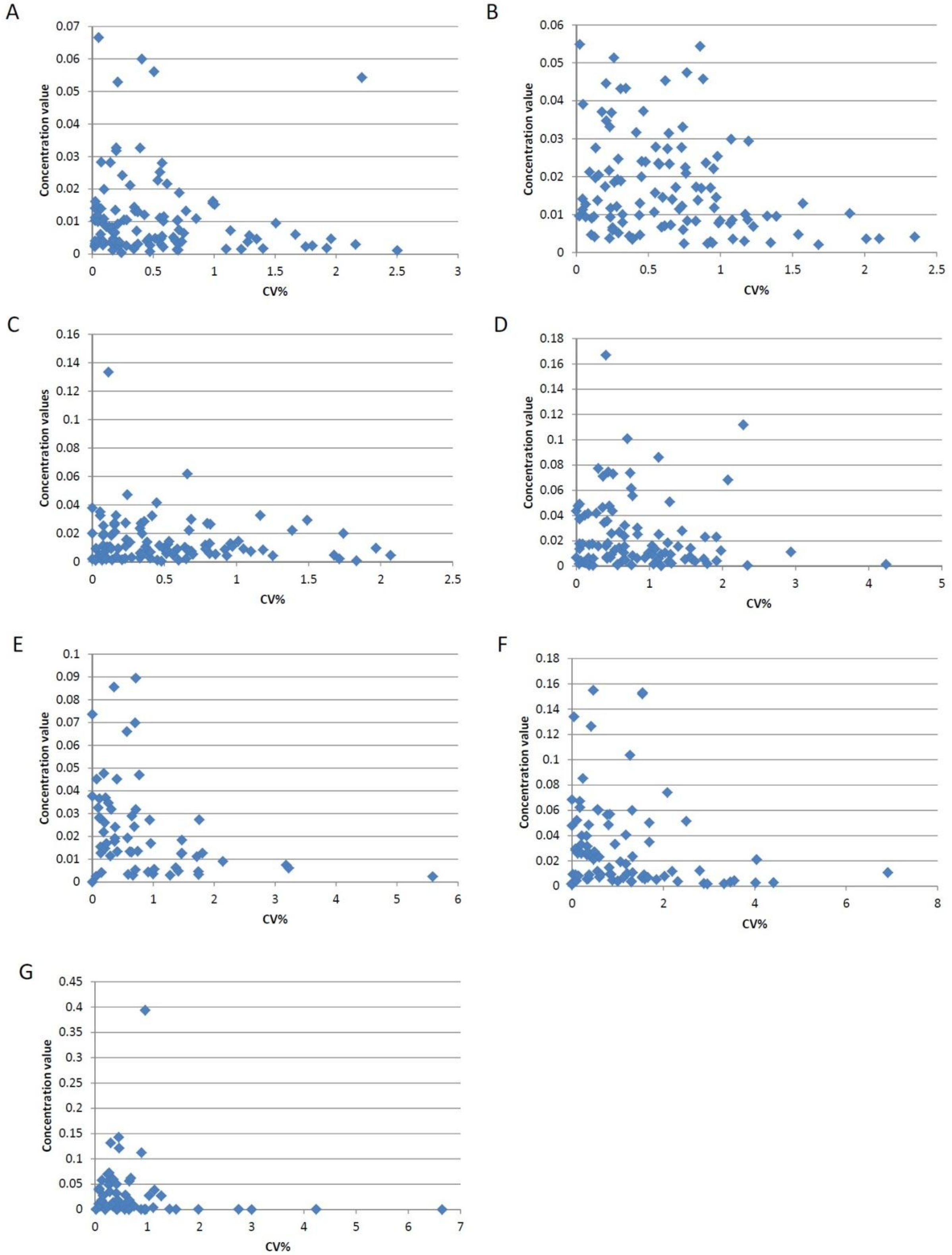

Figure S3. The scatterplots presenting CV% and concentration values of leaf samples. The CV% ($SD_{Cq}/mean_{Cq}$) values were calculated from two technical replicates of each utilized primer pair and the concentration values were generated with Abs Quant/2nd Derivative Max for All Samples Analysis of Lightcycler 480 Software release 1.5.0 SP3. A, *Atub*; B, *PP2A*; C, *Bp4CL1*; D, *Bp4CL2*; E, *Bp4CL3*; F, *Bp4CL4*; G, *Pt4CL1*.

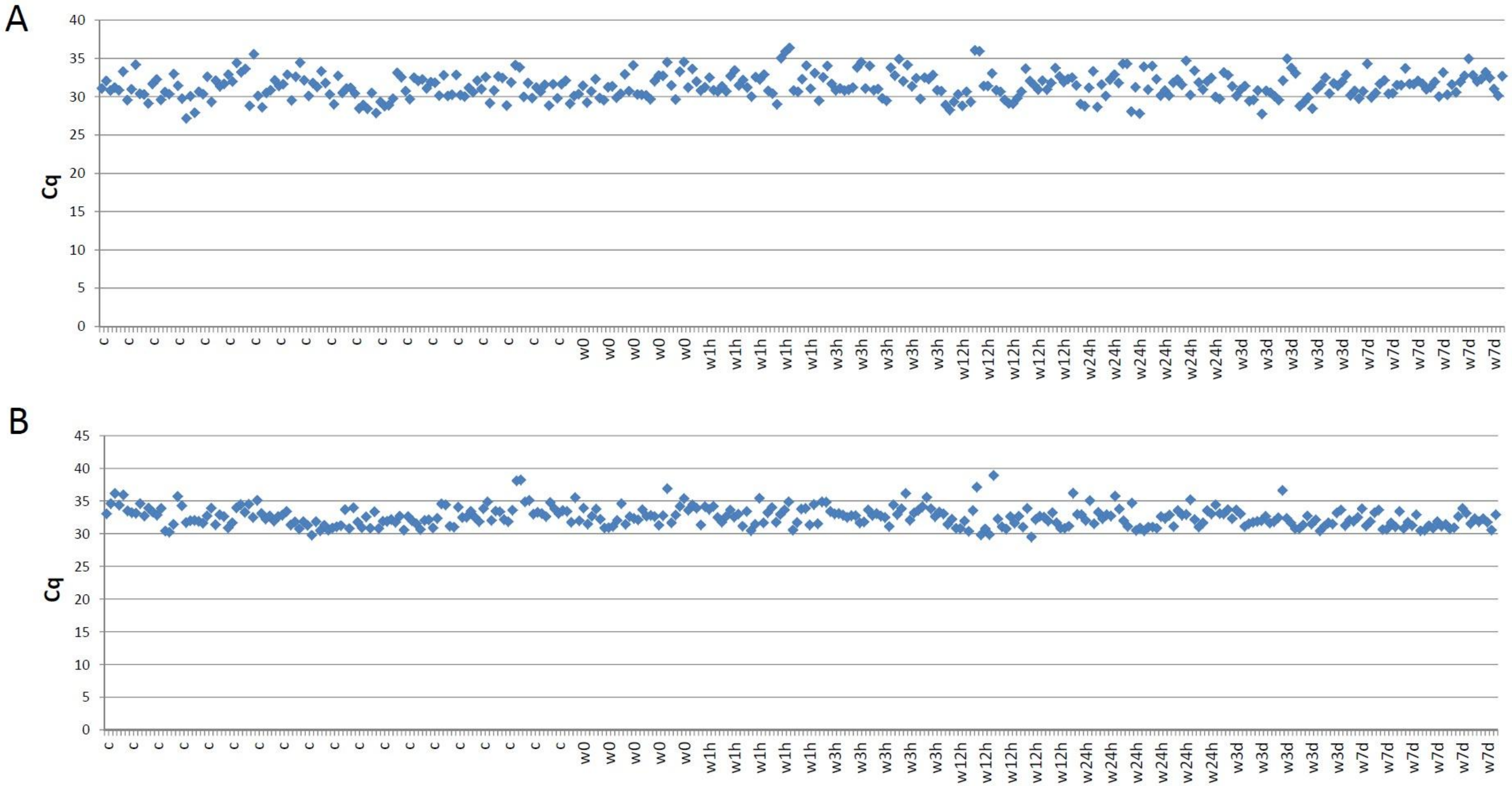

Figure S4. The Cq values of *Atub* and *PP2A* in undamaged and wounded leaves. The Cq values represent crossing point values generated with Abs Quant/2nd Derivative Max for All Samples Analysis of Lightcycler 480 Software release 1.5.0 SP3 of *Atub* (A) and *PP2A* (B) in non-treated leaves (c) and leaves collected immediately after mechanical wounding (w0) and 1 (w1h), 3 (w3h), 12 (w12h), 24 (w24h), 72 (w3d) and 168 (w7d) hours after the wounding treatment.

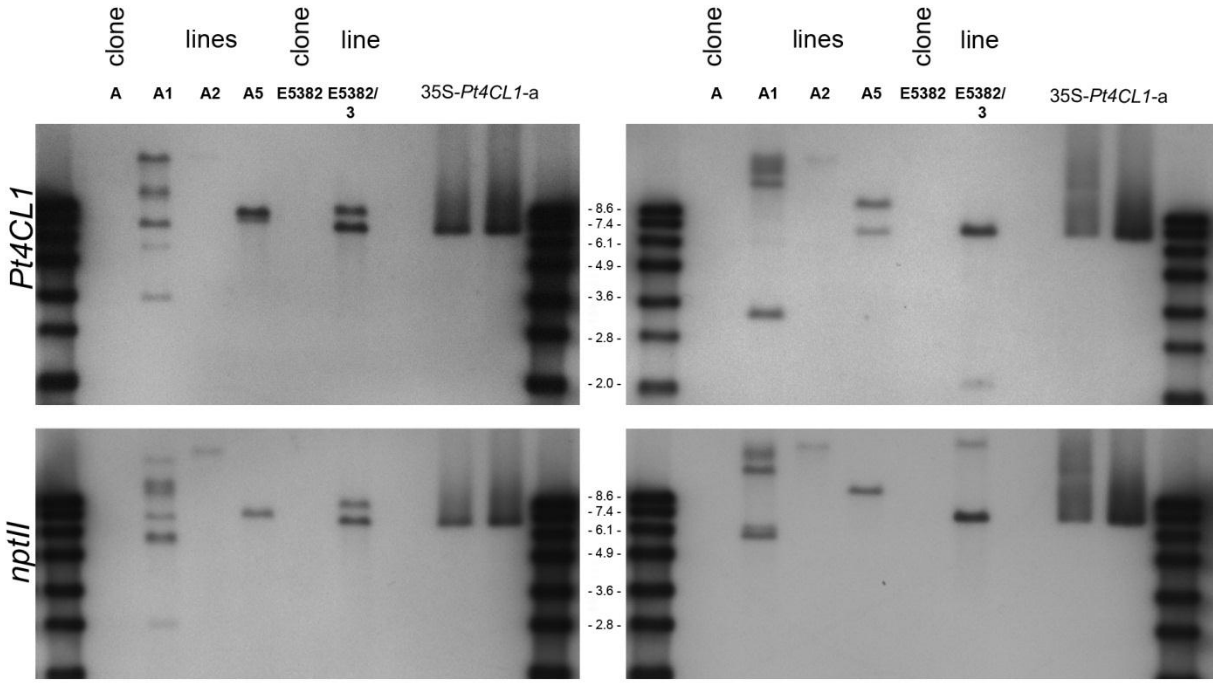

Figure S5. Southern blot analysis of regenerated silver birch lines. Lines A1, A2, A5 and E5382/3 were transformed with pRT99/35S-*Pt4CL1*-a plasmid vector and probed for the presence of the 35S-*Pt4CL1* and *nptII*. A genomic DNA sample of 15 µg digested with *Bam*HI (figures on the left) or *Xba*I (figures on the right) was loaded to each lane.

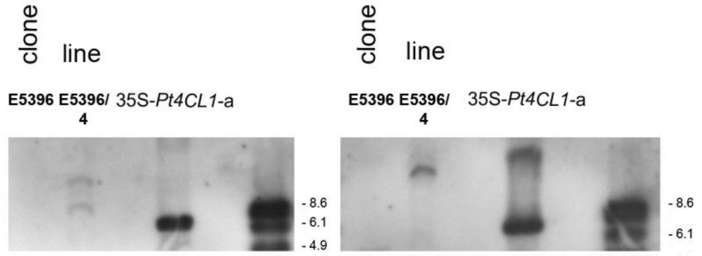

Figure S6. Southern blot analysis of regenerated silver birch line E5396/4. Line was transformed with pRT99/35S-*Pt4CL1*-a plasmid vector and probed for the presence of the *nptII*. A genomic DNA sample of 15 µg digested with *Bam*HI (figures on the left) or *Xba*I (figures on the right) was loaded to each lane.

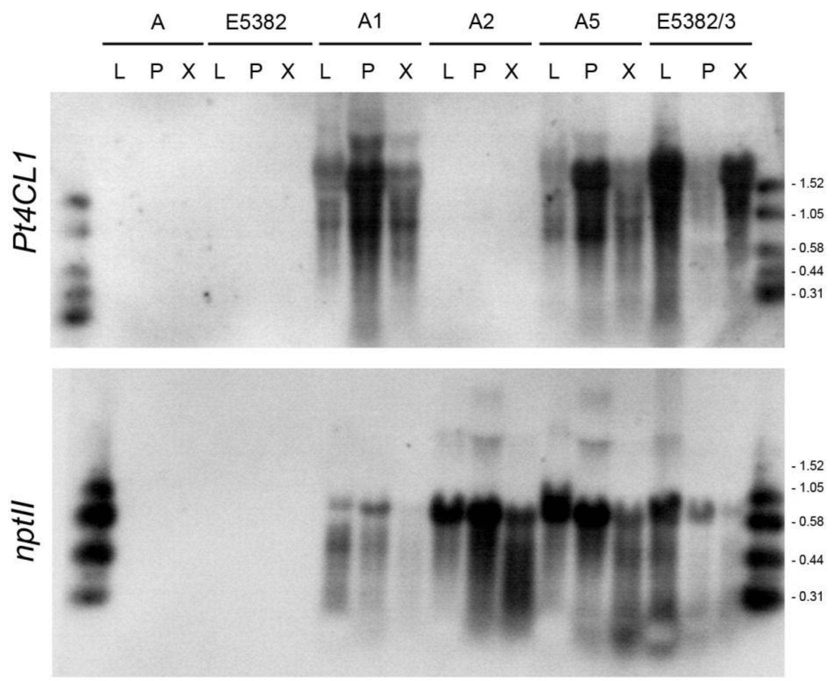

Figure S7. Northern blot analysis of transgenic silver birch lines. RNA samples of 15 µg were isolated from the leaf (L), phloem (P) and xylem (X) of clones A and E5382 and lines A1, A2, A5 and E5382/3. The probes were 1.1 and 0.8 kb for Pt4CL1 and nptII, respectively.

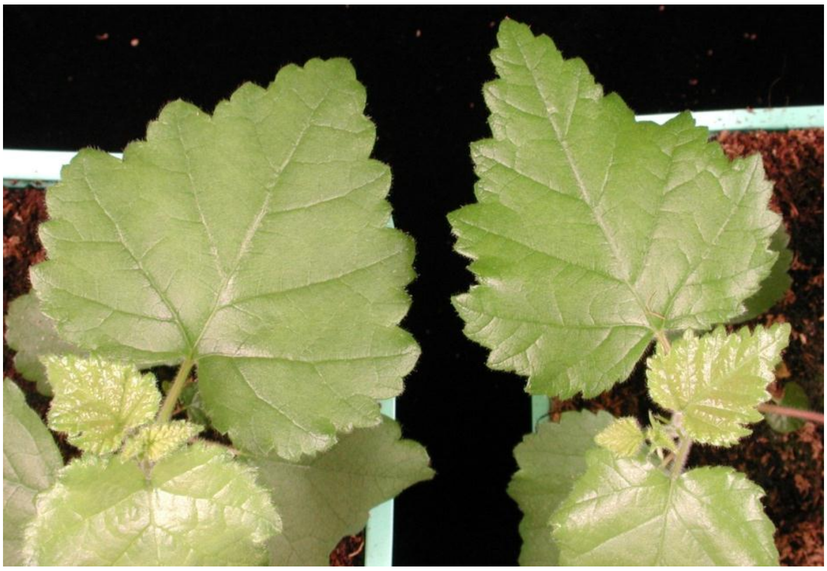

Figure S8. The leaf morphology of Pt4CL1a line E5382/3. During the first growing season in the greenhouse, the leaf margins of Pt4CL1a line E5382/3 (on the left) differed in from clone E5382 (on the right).

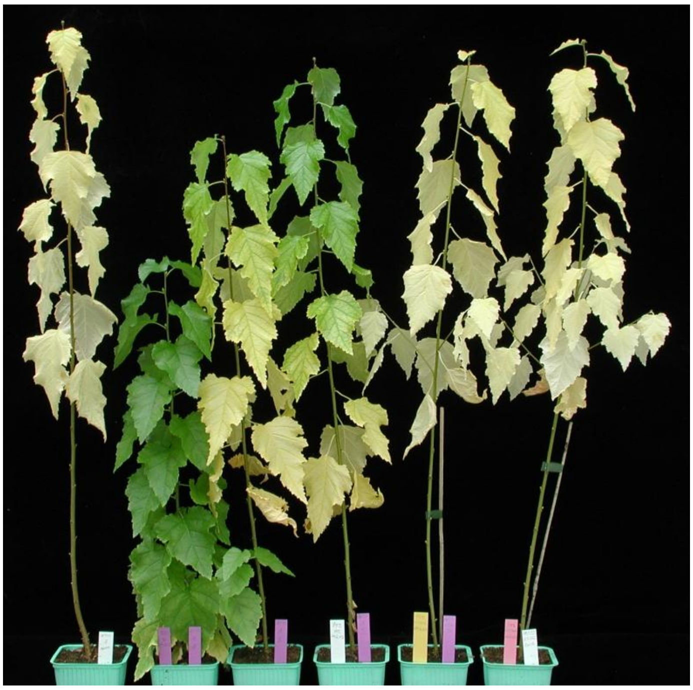

Figure S9. The autumn leaf senescence of silver birch clones and lines. The clone A, line A2, line A5, line E5382/3, and clone E5382 (from left to right) at the end of first growing season at the greenhouse of Finnish Forest Research Institute Punkaharju Unit (photographed on the 6th of October).

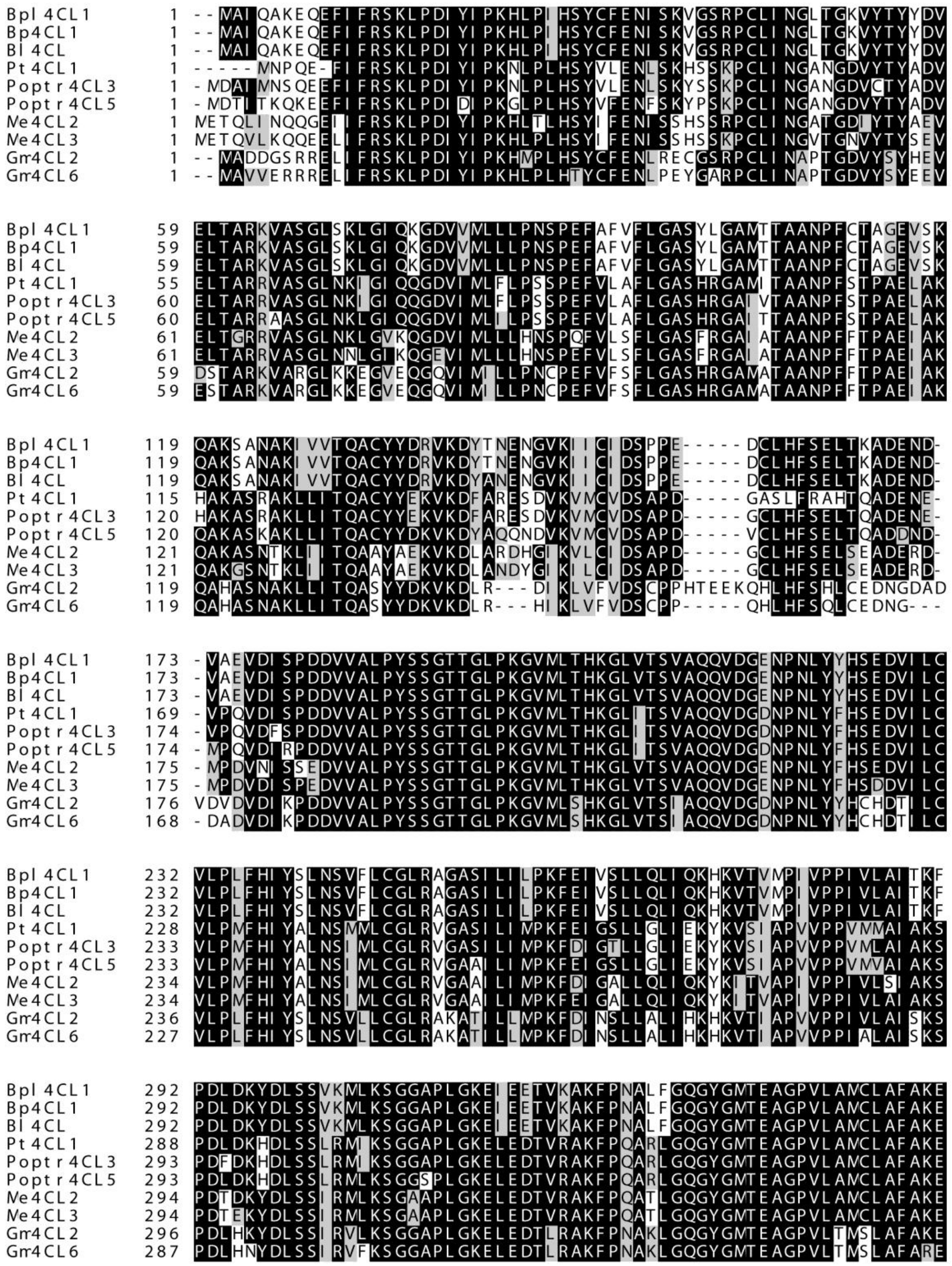

Figure S10. Alignment of predicted amino acid sequence of silver birch (*Betula pendula*) putative 4-coumarate:CoA ligase (Bp4CL1, KM099195). Bp4CL1 aligned with the predicted amino acid sequences of *Betula luminifera* (Bpl4CL1, AY792353), *Betula platyphylla* (Bl4CL, FJ410448), *Glycine max* (Gm4CL2, Glyma13g44950; Gm4CL6, Glyma15g00390), *Manihot esculenta* (Mes4CL2, cassava4.1_005014m; Mes4CL3, cassava4.1_005006m), *Populus tremuloides* (Pt4CL1, AF041049), and *Populus trichocarpa* (Poptr4CL3, grail3.0100002702 LG I; Poptr4CL5, fgenesh4_pg.C_LG_III001773 LG III).

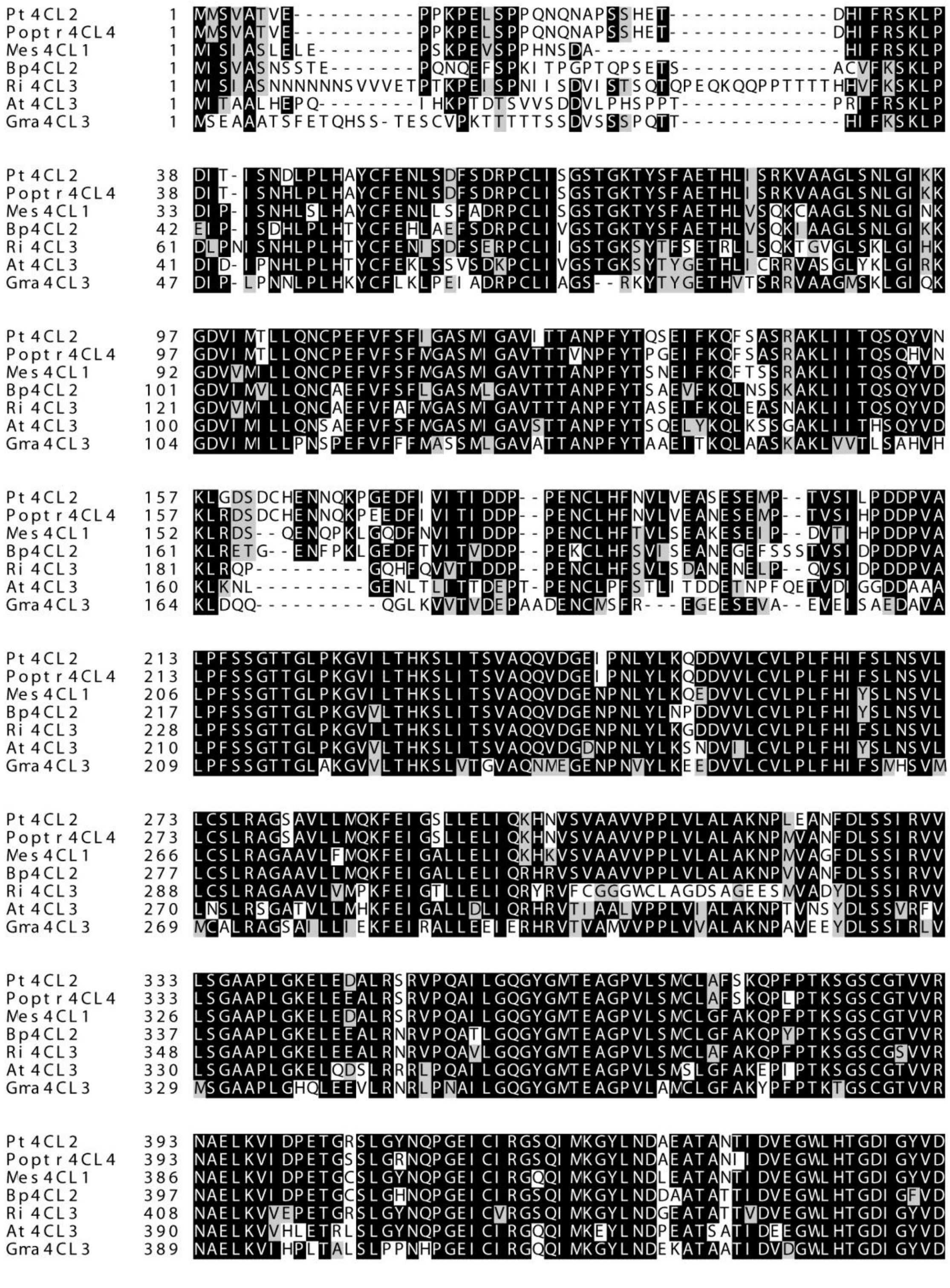

Figure S11. Alignment of predicted amino acid sequence of silver birch (*Betula pendula*) putative 4-coumarate:CoA ligase (Bp4CL2, KM099196). Bp4CL2 aligned with the predicted amino acid sequences of *Arabidopsis thaliana* (At4CL3, At1g65060), *Rubus idaeus* (Ri4CL3, AAF91308), *Glycine max* (Gm4CL3, NM_001250341), *Manihot esculenta* (Mes4CL1, cassava4.1_004658m; Mes4CL4, cassava4.1_004136m), *Populus tremuloides* (Pt4CL2, AF041050), and *Populus trichocarpa* (Poptr4CL4, grail3.0099003002 LG IX).

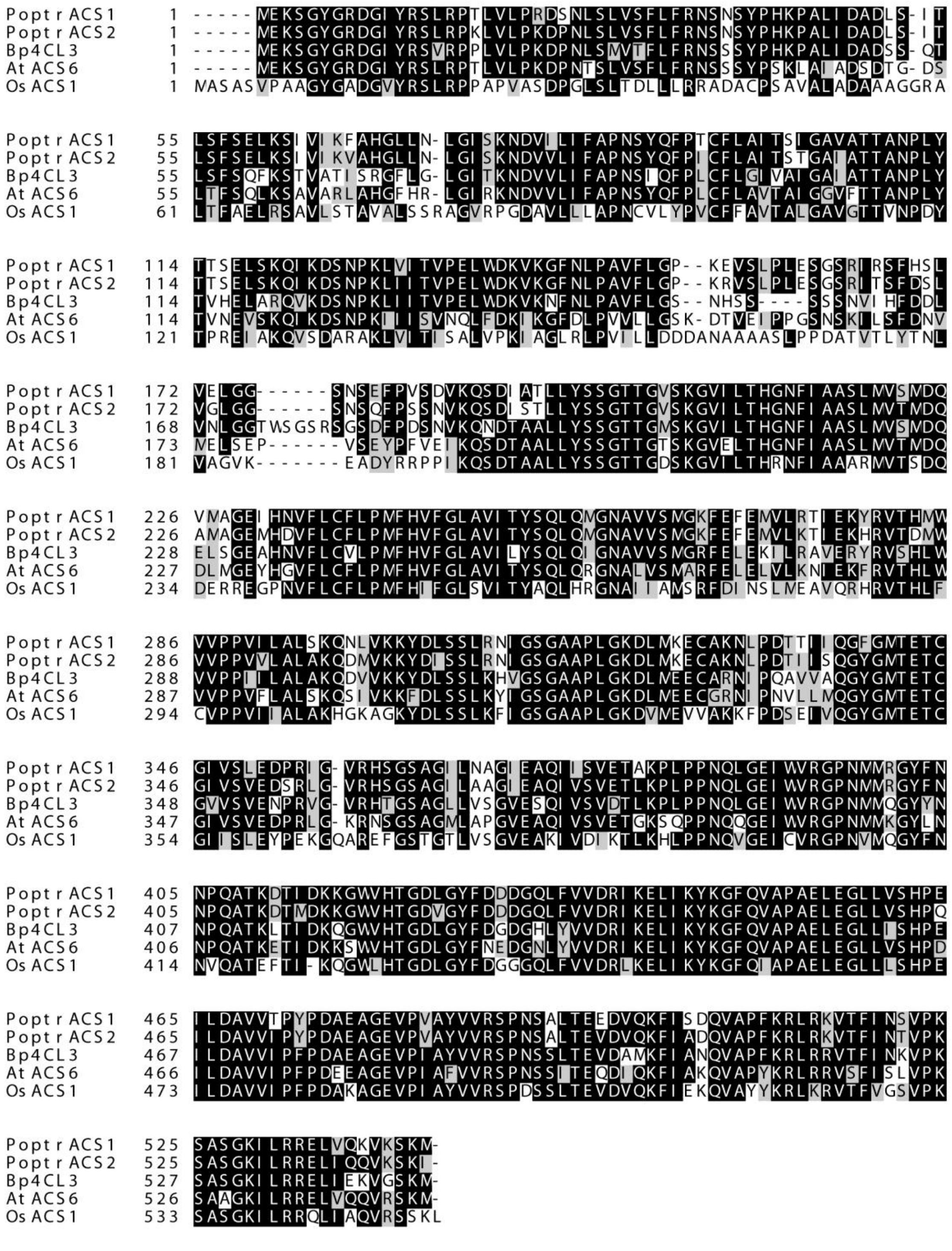

Figure S12. Alignment of predicted amino acid sequence of silver birch (*Betula pendula*) putative 4CL-like acyl-CoA synthetase (ACS) (Bp4CL3, KM099197). Bp4CL3 aligned with the predicted amino acid sequences of *Arabidopsis thaliana* (AtACS6, At4g05160), *Oryza sativa* (OsACS1, Os03g05780) and *Populus trichocarpa* (PoptrACS1, eugene3.01230068; PoptrACS2, estEXT_fgenesh1_pg_v1.C_LG_IV0024).

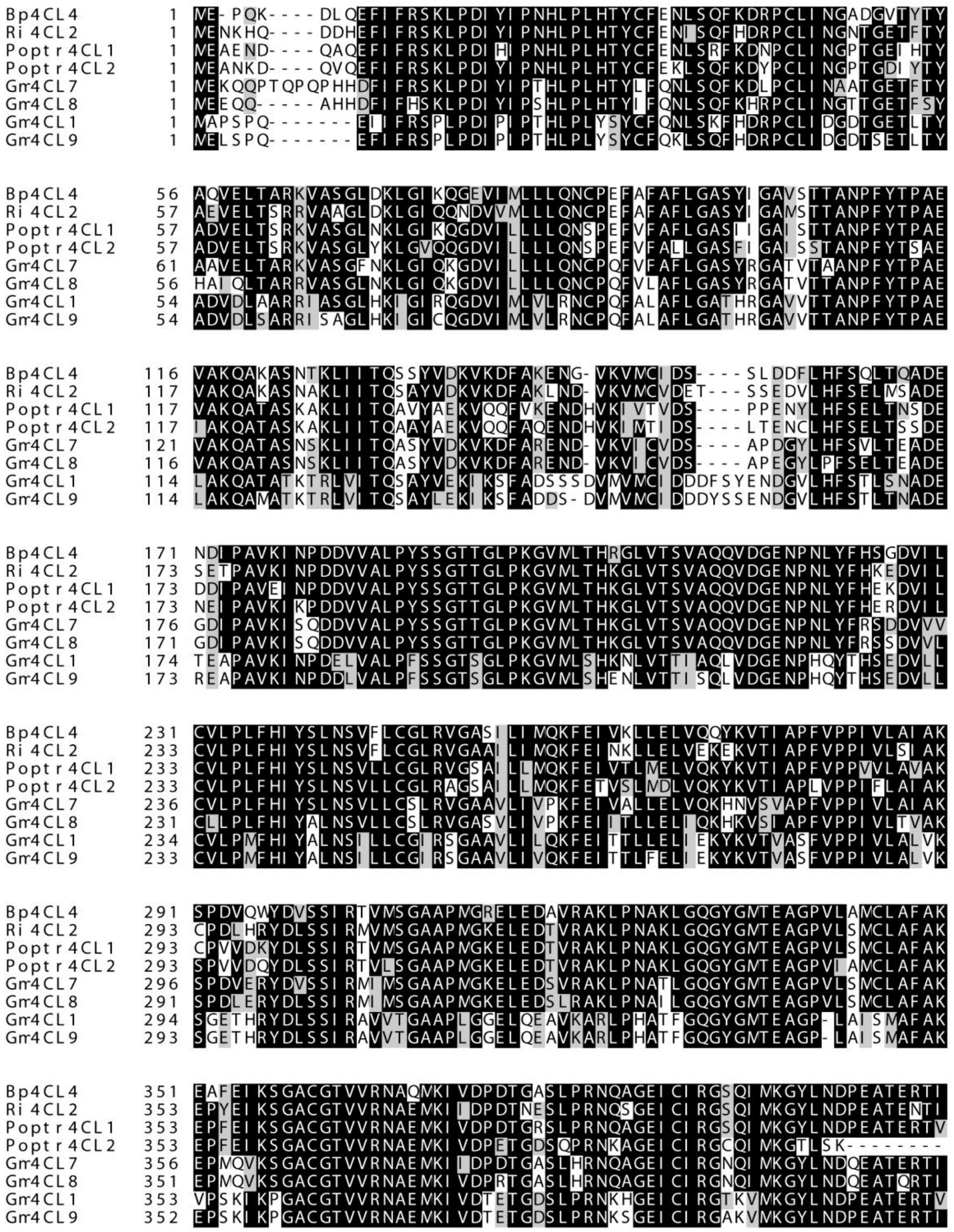

Figure S13. Alignment of predicted amino acid sequence of silver birch (*Betula pendula*) putative 4-coumarate:CoA ligase (Bp4CL4, KM099198). Bp4CL4 with the predicted amino acid sequences of *Rubus idaeus* (Ri4CL2, AAF91309), *Glycine max* (Gm4CL7, Glyma17g07170; Gm4CL8, Glyma17g07180; Gm4CL1, Glyma17g07190; Gm4CL9, Glyma13g01080), and *Populus trichocarpa* (Poptr4CL1, estExt_fgenesh4_pg.C_1210004 scaffold3; Poptr4CL2, gw1.XVIII.2818.1 LG_XVIII).

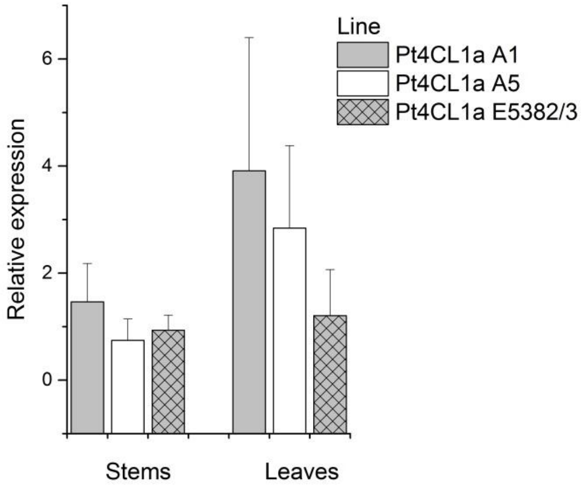

Figure S14. Relative expression of *Pt4CL1* in stems and leaves of Pt4CL1a lines A1, A5 and E5282/3. Values represent means and standard deviations calculated using *PP2A* gene as reference gene (n = 3–14).

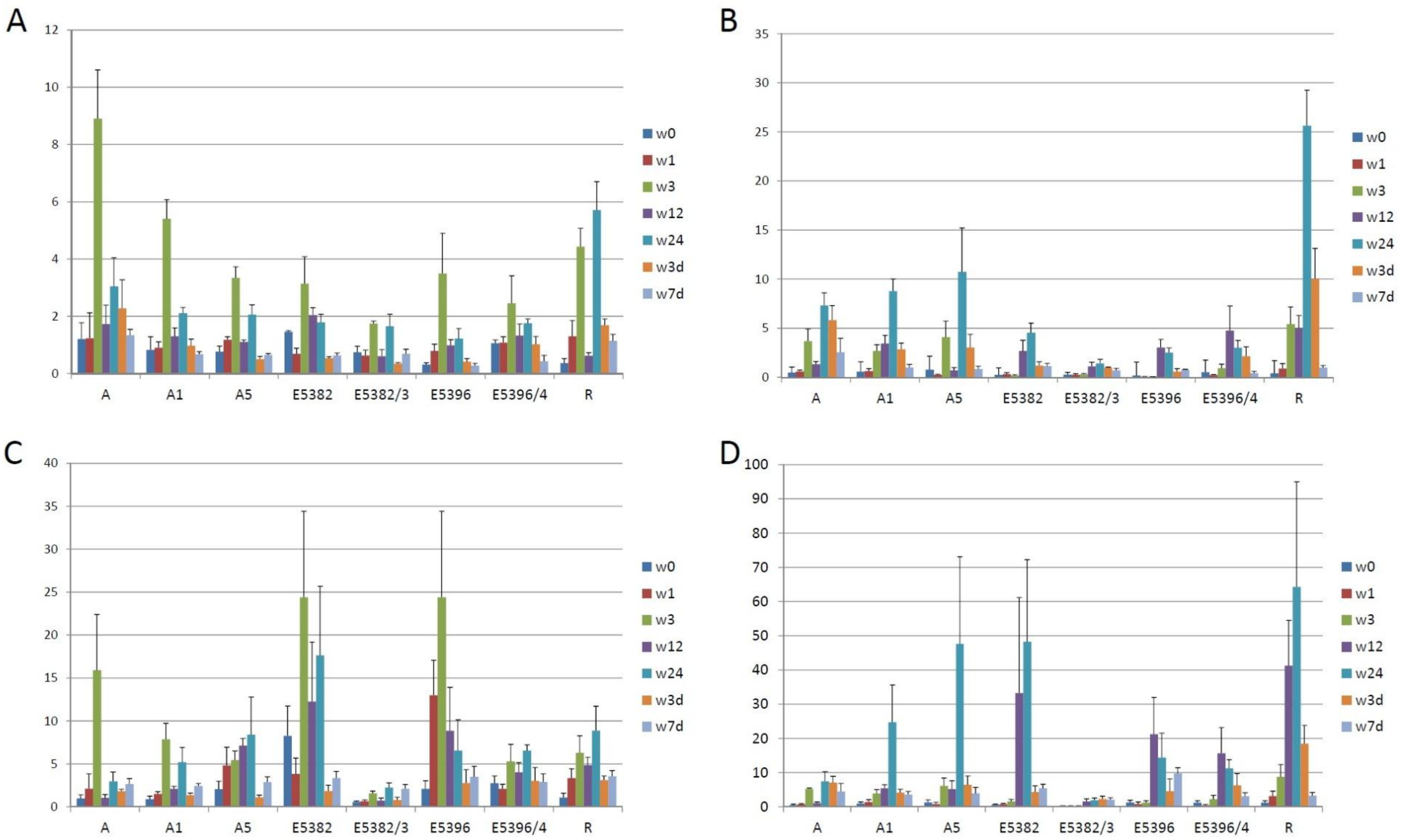

Figure S15. Relative expression of *Bp4CL1-4* genes in mechanically wounded silver birch (*Betula pendula*) leaves. The *Bp4CL1* (A, C) and *Bp4CL2* (B, D) expression in leaves collected immediately (w0) and 1 (w1), 3 (w3), 12 (w12), 24 (w24), 72 (w3d), and 162 (w7d) h after mechanical wounding of clones A, E5382, E5396 and R and transgenic lines A1, A5, E5382/3 and E5396/4. Values represent means and standard errors calculated from the target/refence ratios. A and C, *PP2A* was used as the reference gene; B and D, *Atub* was used as the reference gene.

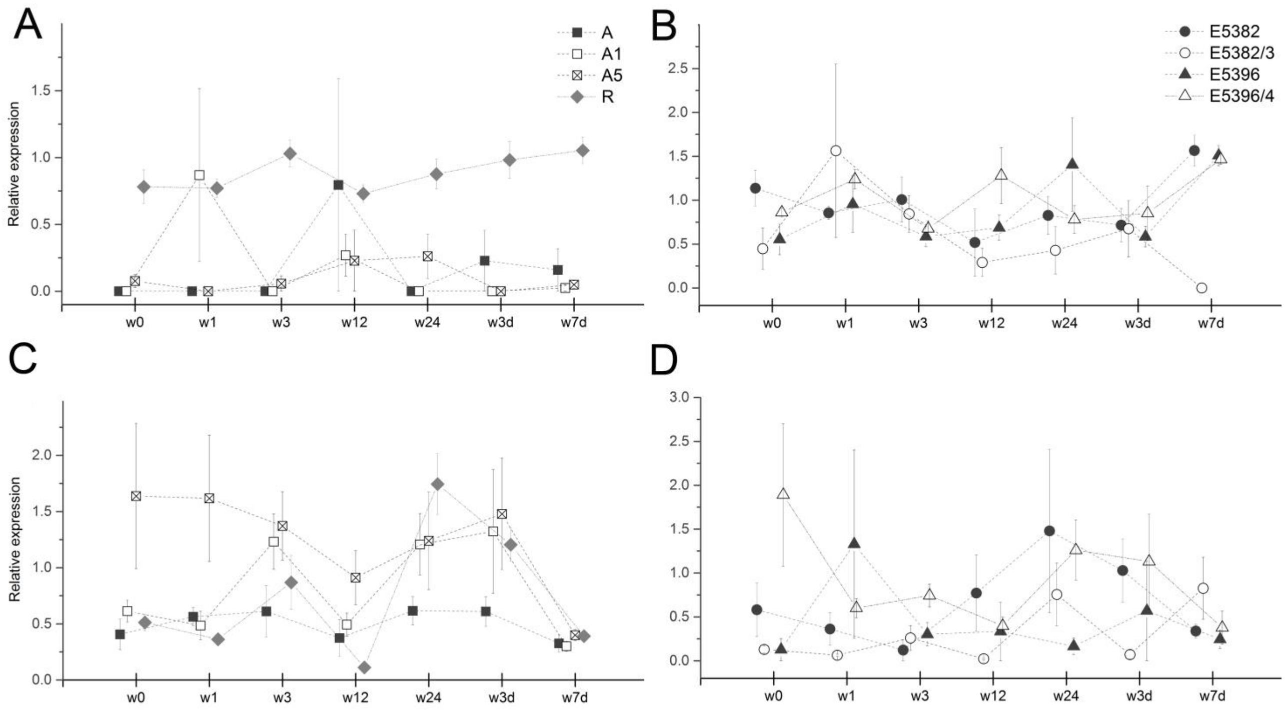

Figure S16. Relative expression of *Bp4CL3* (A, B), and *Bp4CL4* (C, D) in wounded leaves. The leaves were collected immediately (w0) and 1, 3, 12, 24, 48, and 162 h after mechanical wounding of silver birch (*Betula pendula*) clones A and R and transgenic lines A1 and A5 (A, C) and clones E5382 and E5396 and transgenic lines E5382/3 and E5396/4 (B, D). Values represent means and standard errors calculated using the mean of control leaves as normalizer within each line/clone and *PP2A* as reference.
