# Supplementary material for: Phenolic Compounds and Expression of 4CL Genes in Silver Birch Clones and Pt4CL1a Lines
Source: PLoS One. 2014 Dec 11;9(12):e114434. doi: 10.1371/journal.pone.0114434 (PMC4263613; doi:10.1371/journal.pone.0114434)
Supplement: S2 File — This file contains S1-S2 Tables. S1 Table. The primers used in the production the probes, subcloning of cds of putative Bp4CL1-4 and real-time RT-PCR. S2 Table. The performance of Atub and PP2A primers used in the amplification of reference genes. (PDF) [file pone.0114434.s002.pdf]

Table S1. The primers used in the production the probes, subcloning of cds of putative *Bp4CL1-4* and real-time RT-PCR.

Gene	Forward primer (5'→3')	Reverse primer (5'→3')	Product size (bp)	Efficiency stems#	Linear dynamic range (stems)*	Efficiency leaves#	Linear dynamic range (leaves)*
Primers used in the generation of Northern and Southern probes							
Pt4CL1 1)	GGGTTTCAGGTTGCTCCTAC	AGTCTCAGAAGACCACAGGG	892				
2)	TTTGCTCTCGAGGCCTTGGCA	ATCGCAAGACCGGCAACAGG	509				
3)	AACCAGTGTGGCTCAACAGG	TTGCCTGATGGTGCCTTGGG	956				
nptII	TGGGCACAACAGACAATCGG	CAGCAATATCACGGGTAGCC	800				
Primers used in the subcloning of the full cds of putative *4CL*s							
Bp4CL1	CCCCCATCTCTTTGTGCTTA	ATAAAAGGTGTCGTGGGGAAT	1803				
Bp4CL2	CCACACACAATACCCCTAGAAAC	GAACATTGGTTGAGATGGATGA	1912				
Bp4CL3	AATGGAAAAATCAGGGTACGG	AAACATTCAATTTCACATTTTGGA	1652				
Bp4CL4	CAGCAGCCAAACAAACATCA	GGTGATTCCACAAAGCAACA	1797				
Primers used in the real-time RT-PCR							
Bp4CL1	AAGGAGTTGAGGGCAAAA	AAGGTGTCGTGGGGAAT	180	1.8	22.2–28.5	2.0	23.9–29.9
Bp4CL2	CCACACACAATACCCCTAGAAAC	GCTCAAAGCAGTAGGTGTGAAGT	240	1.8	30.2–34.1	1.9	27.1–33.1
Bp4CL3	ACGTGGTGAAAAAGTACGATCTCT	CTAAGCCGAACACCTACTCTTGG	188	2.0	29–35.6	1.9	27.7–34.9
Bp4CL4	TCCTACGTTGACAAAGTGAAAGAC	CGTCATCCGGGTTGATTTTA	152	1.8	28.2–34.7	1.8	32.2–40
Pt4CL1	TCCCCTGCATTCATACGTTC	CCTAGGAAAGCAAGCACGAA	213	1.9	26.3–32.9	1.9	24.9–31

1) Primers used in the generation of probe for *Bam*HI digested genomic DNA (Southern blot), forward primer specific for Pt4CL1, reverse primer specific for 35S promoter

2) Primers used in the generation of probe for *Xba*I digested genomic DNA (Southern blot), forward primer specific for Pt4CL1, reverse primer specific for NOS terminator

3) Primers used in the generation of probe for Northern blot analysis

#The efficiency of primer pair specific calibration curves calculated using the Abs Quant/2nd Derivative Max for All Samples in Lightcycler® 480 Software release 1.5.0 SP3

*The linear dynamic range represents the range of the Cq values between the highest and the lowest concentration of the primer pair specific calibration curve

Table S2. The performance of *Atub* and *PP2A* primers used in the amplification of reference genes.

Gene	Study	Product size (bp)	Material	Growing conditions	Cq^ mean	Cq^ SD	CV%[§] mean	Efficiency#	Linear dynamic range*
Atub	Present study	174	stems	greenhouse	24.2	1.0	0.23	1.8	21.7–27.7
	Present study		leaves	greenhouse	31.1	1.59	0.54	1.8	26.9–33.7
	Unpublished data		leaves	growth chamber	30.1	2.0	0.53	2.0	27.8–34.1
	Unpublished data		leaves	field	28.7	0.93	0.31	2.0	27.3–32.5
	Sutela et al. 2009		roots	greenhouse	30.3	2.4	1.22	2.0	23.1–29.5
PP2A	Present study	213	stems	greenhouse	29.3	1.68	0.55	1.9	26.3–32.9
	Present study		leaves	greenhouse	32.7	1.46	0.76	1.9	24.9–31
	Unpublished data		leaves	growth chamber	34.8	1.7	1.55	2.0	32.9–37.3
	Unpublished data		leaves	field	33.0	1.4	0.72	2.0	30.9–34.9
	Sutela et al. 2009		roots	greenhouse	33.5	1.57	2.16	2.0	26.4–35

^The Cq values represent the crossing points generated with Abs Quant/2nd Derivative Max for All Samples in Lightcycler® 480 Software release 1.5.0 SP3 across the whole dataset of untreated (control) samples

[§]The mean of CV% values of technical replicates across the dataset of untreated (control) samples

#The efficiency of primer pair specific calibration curves calculated using the Abs Quant/2nd Derivative Max for All Samples in Lightcycler® 480 Software release 1.5.0 SP3

*The linear dynamic range represents the range of the Cq values between the highest and the lowest concentration of the primer pair specific calibration curve
