# Supplementary material for: Phenolic Compounds and Expression of 4CL Genes in Silver Birch Clones and Pt4CL1a Lines
Source: PLoS One. 2014 Dec 11;9(12):e114434. doi: 10.1371/journal.pone.0114434 (PMC4263613; doi:10.1371/journal.pone.0114434)
Supplement: S4 File — This file contains S6–S8 Tables. S6 Table. The growth characteristics of silver birch non-transgenic clones and transgenic lines. S7 Table. The phenology of autumn leaf senescence at the end of first growing season. S8 Table. Relative growth rates (mg/d) of lepidopteran larvae. The leaves of silver birch clones (A and E5382) and lines (A1, A2, A5 and E5382/3) were offered to the larvae of Aethalura punctulata, Cleora cinctaria, Epirrita autumnata, and Orthosia gothica. (PDF) [file pone.0114434.s004.pdf]

**Table S6. The growth characteristics of silver birch non-transgenic clones and transgenic lines.**

| Clone / Line | 1st growing season | 2nd growing season | 3rd growing season |
|---|---|---|---|
| Stem height (m) | | | |
| A | 0.94 ± 0.1 (51) | 1.86 ± 0.13 (44) | 2.41 ± 0.12 (9) |
| A1 | 0.77 ± 0.09 (51) *** | 1.7 ± 0.09 (41) *** | 2.1 ± 0.05 (9) ** |
| A2 | 0.58 ± 0.08 (51) *** | 1.49 ± 0.13 (40) *** | 1.93 ± 0.09 (9) ** |
| A5 | 0.88 ± 0.09 (51) *** | 1.85 ± 0.15 (44) | 2.4 ± 0.01 (9) |
| E5382 | 0.84 ± 012 (51) | 1.74 ± 0.1 (44) | 2.08 ± 0.11 (9) |
| E5382/3 | 0.91 ± 0.11 (43) ** | 1.82 ± 0.11 (35)*** | 2.16 ± 0.06 (9) |
| Stem diameter (mm) | | | |
| A | NA | 11.17 ± 0.92 (44) | 12.77 ± 0.96 (9) |
| A1 | NA | 10.05 ± 0.76 (41) *** | 13.17 ± 0.92 (9) |
| A2 | NA | 9.16 ± 1.23 (40) *** | 12.8 ± 1.06 (9) |
| A5 | NA | 11.0 ± 0.89 (44) | 13.73 ± 1.22 (9) |
| E5382 | NA | 9.36 ± 0.86 (44) | 11.73 ± 0.67 (9) |
| E5382/3 | NA | 9.62 ± 0.86 (35) | 11.86 ± 0.5 (9) |
| Stem height (m) | | | |
| E5396 | 0.14 ± 0.04 (60) | 1.2 ± 0.27 (60) | NA |
| E5396/4 | 0.04 ± 0.01 (60) *** | 0.2 ± 0.11 (60) *** | NA |
| Stem diameter (mm) | | | |
| E5396 | 3.73 ± 0.46 (60) | 7.5 ± 0.87 (60) | NA |
| E5396/4 | 2.58 ± 0.33 (60) *** | 3.54 ± 0.56 (60) *** | NA |

Clone A and E5382 and lines A1, A2, A5 and E5382/3 were taken to greenhouse in June and were in the same experimental set-up for two years, which after the number of individual silver birches per clone/line was reduced to 9. The experimental set-up for clone E5396 and transgenic line E5396/4 was started one year later in August. The growth parameters were recorded in the end of each growing season. Pairwise comparison of non-transgenic clone and transgenic line according to the independent-samples t-test, or Wilcoxon test. The number of biological replicates in parentheses.* = $P < 0.05$, ** = $P < 0.01$, *** = $P < 0.001$, NA = value not recorded

**Table S7. The phenology of autumn leaf senescence at the end of first growing season.**

| Clone / Line | Class | 26 Sept. % | 2 Oct. % | 9 Oct. % |
|---|---|---|---|---|
| A | A | 2 | | |
| | B | 55 | | |
| | C | 39 | 16 | |
| | D | 4 | 84 | 100 |
| A1 | A | 12 | | |
| | B | 84 | 10 | |
| | C | 3 | 61 | |
| | D | | 29 | 100 |
| A2 | A | 73 | 6 | |
| | B | 27 | 82 | 2 |
| | C | | 10 | 39 |
| | D | | 2 | 59 |
| A5 | A | | | |
| | B | 82 | 10 | |
| | C | 18 | 37 | 2 |
| | D | | 53 | 98 |
| E5382 | A | | | |
| | B | 39 | | |
| | C | 51 | 14 | |
| | D | 10 | 86 | 100 |
| E5382/3 | A | 2 | | |
| | B | 28 | | |
| | C | 58 | 2 | |
| | D | 12 | 98 | 100 |

The yellowing of leaves was categorised to four classes at three dates (the 26$^{th}$ September, the 2$^{nd}$ of October, and the 9$^{th}$ of October). The classes were A) leaves are green; B) some yellowing leaves; C) less than 50% of leaves are yellowing; D) more than 50% of leaves are yellowing. Values represent percentages of plants falling to each class at separate dates within each clone/line.

**Table S8. Relative growth rates (mg/d) of lepidopteran larvae.** The leaves of silver birch (*Betula pendula*) clones (A and E5382) and lines (A1, A2, A5 and E5382/3) were offered to the larvae of *Aethalura punctulata*, *Cleora cinctaria*, *Epirrita autumnata*, and *Orthosia gothica*.

| | | Clone | Lines | | | Clone | Line |
|---|---|---|---|---|---|---|---|
| Species | | A | A1 | A2 | A5 | E5382 | E5382/3 |
| *A. punctulata* | I | 0.26 ± 0.12 (13) | 0.36 ± 0.1 (14) | 0.29 ± 0.12 (15) | 0.29 ± 0.07 (14) | 0.32 ± 0.12 (15) | 0.27 ± 0.10 (14) |
| | II | 0.35 ± 0.2 (14) | 0.29 ± 0.12 (12) | 0.32 ± 0.11 (14) | 0.30 ± 0.08 (14) | 0.29 ± 0.1 (14) | 0.29 ± 0.1 (15) |
| *C. cinctaria* | I | 0.42 ± 0.09 (15) | 0.39 ± 0.17 (12) | 0.40 ± 0.17 (14) | 0.40 ± 0.12 (10) | 0.46 ± 0.1 (15) | 0.49 ± 0.14 (13) |
| | II | 0.37 ± 0.13 (14) | 0.43 ± 0.18 (11) | 0.44 ± 0.15 (13) | 0.38 ± 0.19 (12) | 0.35 ± 0.18 (12) | 0.35 ± 0.16 (16) |
| *E. autumnata* | I | 0.25 ± 0.06 (15) | 0.28 ± 0.1 (11) | 0.27 ± 0.11 (12) | 0.22 ± 0.11 (10) | 0.21 ± 0.1 (12) | 0.16 ± 0.12 (10) |
| | II | 0.23 ± 0.16 (13) | 0.26 ± 0.06 (10) | 0.28 ± 0.12 (10) | 0.30 ± 0.13 (11) | 0.28 ± 0.13 (13) | 0.18 ± 0.12 (15) |
| *O. gothica* | | 0.40 ± 0.27 (10) | 0.46 ± 0.28 (13) | 0.51 ± 0.28 (8) | 0.50 ± 0.27 (12) | 0.44 ± 0.3 (12) | 0.50 ± 0.27 (9) |

Values represent RGR mean (±SD, number of biological replicates in parentheses).
